# *Staphylococcus aureus* infection dynamics

**DOI:** 10.1371/journal.ppat.1007112

**Published:** 2018-06-14

**Authors:** Eric J. G. Pollitt, Piotr T. Szkuta, Nicola Burns, Simon J. Foster

**Affiliations:** Department of Molecular Biology and Biotechnology, Firth Court, University of Sheffield, Western Bank, Sheffield, United Kingdom; Columbia University, UNITED STATES

## Abstract

*Staphylococcus aureus* is a human commensal that can also cause systemic infections. This transition requires evasion of the immune response and the ability to exploit different niches within the host. However, the disease mechanisms and the dominant immune mediators against infection are poorly understood. Previously it has been shown that the infecting *S*. *aureus* population goes through a population bottleneck, from which very few bacteria escape to establish the abscesses that are characteristic of many infections. Here we examine the host factors underlying the population bottleneck and subsequent clonal expansion in *S*. *aureus* infection models, to identify underpinning principles of infection. The bottleneck is a common feature between models and is independent of *S*. *aureus* strain. Interestingly, the high doses of *S*. *aureus* required for the widely used “survival” model results in a reduced population bottleneck, suggesting that host defences have been simply overloaded. This brings into question the applicability of the survival model. Depletion of immune mediators revealed key breakpoints and the dynamics of systemic infection. Loss of macrophages, including the liver Kupffer cells, led to increased sensitivity to infection as expected but also loss of the population bottleneck and the spread to other organs still occurred. Conversely, neutrophil depletion led to greater susceptibility to disease but with a concomitant maintenance of the bottleneck and lack of systemic spread. We also used a novel microscopy approach to examine abscess architecture and distribution within organs. From these observations we developed a conceptual model for *S*. *aureus* disease from initial infection to mature abscess. This work highlights the need to understand the complexities of the infectious process to be able to assign functions for host and bacterial components, and why *S*. *aureus* disease requires a seemingly high infectious dose and how interventions such as a vaccine may be more rationally developed.

## Author summary

*Staphylococcus aureus* is a major human pathogen that causes a wide variety of infections. In animal infection models, high doses of *S*. *aureus* are generally required to establish infections. We have recently shown in animal models that this is due to very few bacteria within the infecting population going on to cause disease. This population bottleneck during infection is an ideal target for treatment development. Here we have shown that this bottleneck is common to a range of different *S*. *aureus* types and infection models. The mouse survival model is commonly used for testing antistaphylococcal treatments. We show that the large infectious dose it requires diminishes the bacterial population bottleneck. This suggests the host immune system is simply overwhelmed and brings into question the applicability of such a model. We found that both macrophages and neutrophils are important for resistance to *S*. *aureus* infection but these affect the population bottleneck differently. Macrophages in the liver mediate the initial population bottleneck whereas neutrophils enable the subsequent spread of bacteria to other organs. This has allowed a model of *S*. *aureus* disease progression to be established and our study gives insights into how novel treatments could be established to counter it.

## Introduction

*Staphylococcus aureus* is a leading opportunistic human pathogen renowned for its ability to evade the immune system and cause a variety of different infections [1]. *S*. *aureus* infections can vary from superficial skin lesions, through deep seated abscesses to life threatening sepsis [1]. The diversity of disease modalities has made an understanding of the underlying principles of infection challenging. *S*. *aureus* primarily occurs as a human commensal, mostly in the nares from whence it is able to seed infection. Many *S*. *aureus* infections are iatrogenic, and these are commonly associated with the colonisation of indwelling medical devices [2]. Typically during an infection, after invasion, an immune reaction is initiated by macrophages and these release cytokines to summon neutrophils [3]. Fibrosis also occurs, as well as the death of many of the invading immune cells leading to the pus filled abscesses associated with *S*. *aureus* infections. *S*. *aureus* can also regularly escape local infection sites and disseminate further. If it enters the bloodstream this can lead to sepsis as well as invasion of other organs whereby further local infections can occur. Thus *S*. *aureus* infection is a highly dynamic process with broad dissemination and repeated metastases.

Phagocytosis by professional phagocytes such as macrophages and neutrophils is the primary mode by which *S*. *aureus* is controlled by the immune system. However, *S*. *aureus* has multiple mechanisms to subvert the immune response [4,5], including the production of a variety of specific toxins, the ability to survive intracellularly within immune cells and the capacity to evade capture by phagocytes. As *S*. *aureus* is effectively ubiquitous in the human environment, our immune systems are exposed to this organism as evidenced by the significant bloodstream titre of antibodies against a variety of *S*. *aureus* antigens [4,6–8], however these do not necessarily afford protection against infection. It is therefore the subject of much debate as to what are the key immune factors that control *S*. *aureus* due to the variety of possible infections as well as the many immune components involved [9,10]. This is of great translational importance as the correlates of protection against infection will determine crucial developments such as a vaccine to prevent disease [9,10]. In order to develop the knowledge base to underpin such an intervention it is necessary to first understand the correlates of disease, which in itself is dependent on mapping disease progression from initial infection to resolution in favour of the host or the pathogen.

To understand the factors that are important for disease progression it is first necessary to determine the population, spatial and temporal dynamics of the infectious agent within the host. This will highlight the immune breakpoints through which the pathogen must pass to achieve a productive infection. A key way to demonstrate this has been through the creation of multiple tagged variant strains of the pathogen of equivalent fitness and passaging them, at specific ratios, through the relevant host model of disease [11–15]. Subsequent analysis of overall pathogen numbers and the ratios of the tagged strains allows determination of the within host population dynamics. Coupling this with temporal and spatial (specific organ) analysis provides a map of the spread and proliferation of the infectious agent, and highlights the potential for population bottlenecks from which clonal expansion may occur. In model animal systems this can also be combined with the manipulation of the host and/or pathogen to determine the role of multiple factors in disease. This type of study has been very revealing for a range of pathogens such as *Streptococcus pneumoniae*, *Yersinia pseudotuberculosis* and *Salmonella enterica* [12,14,16]. We have used this approach with *S*. *aureus* to demonstrate a population bottleneck during systemic infection in murine and zebrafish embryo models [13,17]. An initial discovery in the zebrafish model was used to inform the design of murine experiments. Using an intravenous administration of a mix of 3 antibiotic resistance marker tagged *S*. *aureus* Newman HG (NewHG) clones it was found that the bacteria are initially largely sequestered in the liver and spleen. Bacterial numbers subsequently slowly decreased in the liver with concomitant increasing numbers in the kidneys. In the liver and spleen the initial ratio of strains is apparent but surprisingly there is a significant alteration in strain ratio in the kidneys consistent with individual bacteria founding the characteristic abscesses [13]. This clonal expansion increases in all organs during infection but is most pronounced in the kidneys. These results show that a population bottleneck can occur in the mouse alluding to a metastatic spread of bacteria from a primary site of infection to the kidneys. In zebrafish, manipulation of professional phagocytes demonstrated the population bottleneck to be likely neutrophil mediated (with the involvement of macrophages)[17,18]. Since it is mediated by immune cells we would also define this population bottleneck as an immune bottleneck. This provides an initial framework onto which to map within host population dynamics and the influence of host immune defences.

The basis for key developments in our understanding of disease relies on the use of animal models, the aim of which is to reflect facets of human disease. There are a multiplicity of *S*. *aureus* infection models available, ranging from insects (e.g. *Drosophila* & *Galleria*), to zebrafish and mammals (e.g. mice and rabbits) [9,17,19,20]. Mammalian models are considered the most apposite for *S*. *aureus* infections as they share several important characteristics with humans, for instance body temperature, innate and adaptive immune systems and disease pathology. However mammalian models typically require a high initial inoculum to ensure disease initiation and many *S*. *aureus* virulence determinants are human specific [9,21]. Consequently, it is difficult to equate animal model data with the human situation, particularly in the translational context where for instance, despite promising animal model data, vaccine trials have failed in the clinic [9,10]. Given these caveats, animal models are still important conduits for discovery of basic disease parameters. However it is important to evaluate the relative merits of the available models before relying on them to make useful conclusions.

In this study we have used 3 models of systemic disease: firstly the zebrafish model to provide initial data to inform murine studies and then the mouse sepsis and survival models (15, 18–20). The sepsis model is well established and gives a clear progression of bacterial population dynamics [22]. The survival model has been standard for many studies and is fundamentally similar to the sepsis model in that bacteria are commonly injected in to the bloodstream. However the dose is relatively much higher to cause the subjects to succumb to infection rather than survive and resolve as in the sepsis model [23]. The survival, or lethal challenge model, has been in general use and is of regulatory importance [21,23,24]. Here, we have evaluated infection dynamics in the above systemic models of infection with a suite of otherwise isogenic, antibiotic resistant variants of a range of *S*. *aureus* strains. This has revealed population bottlenecks within organ pathogen spatial distribution and established the role of specific immune effector cells in the dynamics of infection. This has led to the formulation of a new conceptual model for disease progression.

## Results

### *S*. *aureus* population dynamics in zebrafish

To evaluate potential strain-specific and generic pathogenesis parameters the population dynamics of a range of different *S*. *aureus* strains (Newman, NewHG, SH1000 and USA300) in the zebrafish embryo systemic model was first determined. Three matched antibiotic resistant *S*. *aureus* strains in the USA300 (JE2) strain background: erythromycin/lincomycin-resistant, EryR (EPPS1); kanamycin-resistant, KanR (EPPS2); and tetracycline-resistant, TetR (EPPS3), and the Newman strain background EryR (EPPS4), KanR (EPPS5), TetR (EPPS6) were produced by transduction of the relevant markers from the previously constructed NewHG strains [13]. The established zebrafish embryo model of systemic disease was then used [17], whereby a 1:1:1 mixture of the variants of each strain was injected into embryos 30 hours post fertilisation (c. 1500 CFU (colony forming units) in total), followed by monitoring of host survival. Bacteria were harvested from embryos and their total numbers and proportions of each *S*. *aureus* strain variant determined. Each marked variant was equally likely to predominate (group variation was not significantly different, Bartlett’s test for equal variance USA300: p = 0.1252, Newman: p = 0.2364), demonstrating that there were no relative fitness costs associated with the individual antibiotic resistance markers which would undermine the analysis [11] (S1 Fig). The proportion of the different strains was used to generate the species evenness index for the population [25], which defines how evenly matched different populations of organisms are within an environment. A population evenness of 1 for a given population (whether it was a host or organ) means the strain variants are evenly distributed (1:1:1 ratio) whereas 0 means the entire population consists of one strain variant. We chose the population evenness metric as it is a commonly accepted in ecological studies [26,27]. It is suitable as it is based on Shannon’s diversity index which is equally sensitive to very rare and very common species in a sample. Our samples inherently had these properties as the populations varied from evenly mixed to completely dominated by 1 variant.

**Fig 1:**
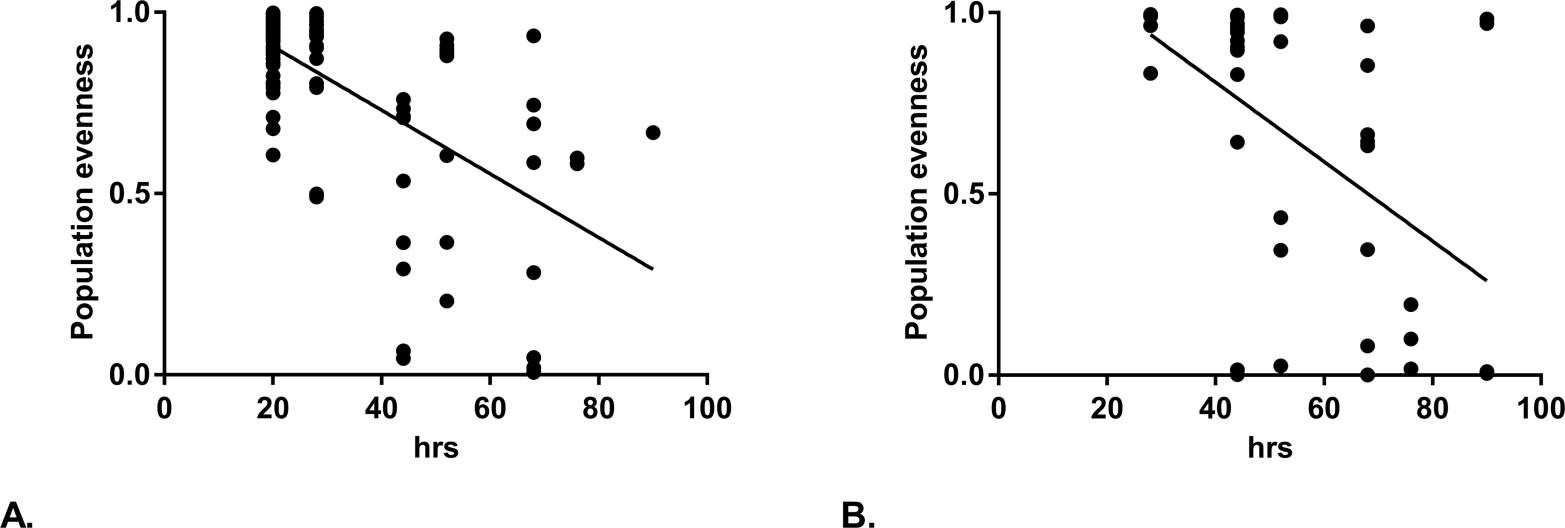
Population evenness (PE) in zebrafish declines over time. Embryos were infected with a 1:1:1 mix of the antibiotic resistance tagged variants. The proportions in each dead fish were analysed and the population evenness determined. (A) Population evenness over the time course of the experiment for USA300. At each time point the following numbers of zebrafish embryos were removed due to reaching severity limits: 20hrs: 49, 28hrs:20, 44hrs:9, 52hrs:8, 68hrs:8, 76hrs:2, 90hrs:1. (B) Population evenness over the time course of the experiment for Newman. PE of 1 is a balanced mixed population whilst PE of 0 is clonal. At each time point the following numbers of zebrafish embryos were removed due to reaching severity limits: 20hrs: 49, 28hrs:6, 44hrs:15, 52hrs:6, 68hrs:9, 76hrs:3, 90hrs:4. Line: mean linear regression. Linear regression, USA300: P<0.0001, F = 57.39, R^2^ = 0.3766, Newman: p = 0.0006, F = 13.69, R^2^ = 0.2504.

We found that the variants which randomly came to predominate readily occurred in both the Newman and USA300 infected hosts, i.e. the population evenness of the injected population started at near 1 and became 0 over the course of the experiment (Fig 1). Additionally, there was a statistically significant correlation between time of death and decreasing population evenness for both strain backgrounds (linear regression, USA300: P<0.0001, F = 57.39, R^2^ = 0.3766, Newman: p = 0.0006, F = 13.69, R^2^ = 0.2504, Fig 1). The decrease in population evenness showed that the population had an increased chance of being clonal over time. This meant that the bacterial population in those zebrafish had passed through a likely population bottleneck. This corresponds to the previous observation of clonality in SH1000 and NewHG in the zebrafish model.

### *S*. *aureus* mouse sepsis population dynamics

**Fig 2:**
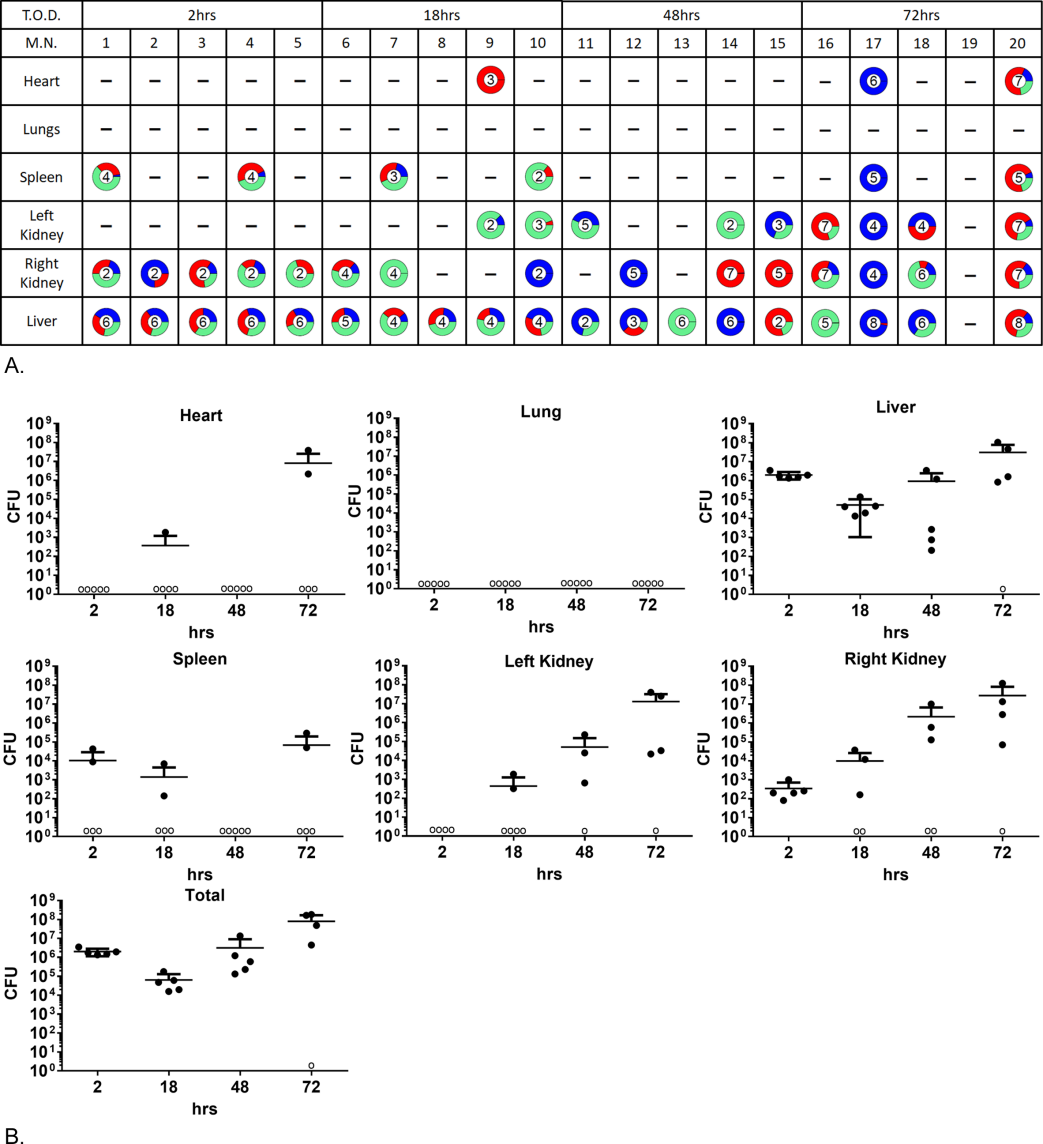
NewHG *S*. *aureus* distribution at different time points during the mouse sepsis model. Mice were infected with a 1:1:1 mixture of 3 resistance marker tagged NewHG variants and 5 mice sacrificed at each time point. (A) The proportions of each strain at each time point in the different organs in each mouse. TetR, EryR and KanR populations are blue, red and green respectively. The number in each pie chart represents the log amount of bacteria present (e.g. 10^−6^ CFU = 6). T.O.D: Time of Death, M.N: Mouse Number. (B) The CFU load at each time point for the organs and total CFU. Organs with CFU counts below the limit of detection (<100CFU) are represented by open circles. Error bars: mean±SD. 5 mice were sacrificed at 2hrs, 18hrs, 48hrs and 72hrs post injection of *S*. *aureus*.

The marked strains were then used for determination of the population dynamics in the established murine sepsis model of infection [22]. A total infectious dose of approximately 1x10^7^ CFU was injected intravenously via the tail for each combination of marked variants in the 4 strain backgrounds (Newman, USA300, SH1000 and NewHG constructed here and previously [13]). Mice were culled at 2, 18, 48 and 72 hours post infection. The heart, lungs, spleen, left and right kidneys and liver were extracted from each subject, homogenised and the CFUs (colony forming units) of the different marked variants determined. As for the zebrafish model the species evenness index was determined. Overall the occurrence of the TetR, EryR and KanR populations was not significantly different in the mice regardless of strain (S1 Fig, Bartlett’s test for equal variance, SH1000: p = 0.5598, NewHG: p = 0.8478, Newman: p = 0.9631 and USA300: p = 0.9330), demonstrating that in all strain backgrounds the antibiotic resistance markers did not impart a fitness cost in the mouse model. The occurrence and distribution of *S*. *aureus* for NewHG, Newman, SH1000 and USA300 are shown in Figs 2 and S2.

#### Total numbers & comparative strain evenness

For all strains, there is an initial drop in the total *S*. *aureus* CFU (2–18 hours) and then a general subsequent increase (Figs 2 and S2). Concomitant with the rise in CFU is a progression towards predomination by particular clones within organs suggesting a common population bottleneck during infection for all strains. Different organs followed specific dynamics and it has been previously shown that within 2 hours *S*. *aureus* CFU drops below detectable limits in the blood [13].

#### Liver

**Fig 3:**
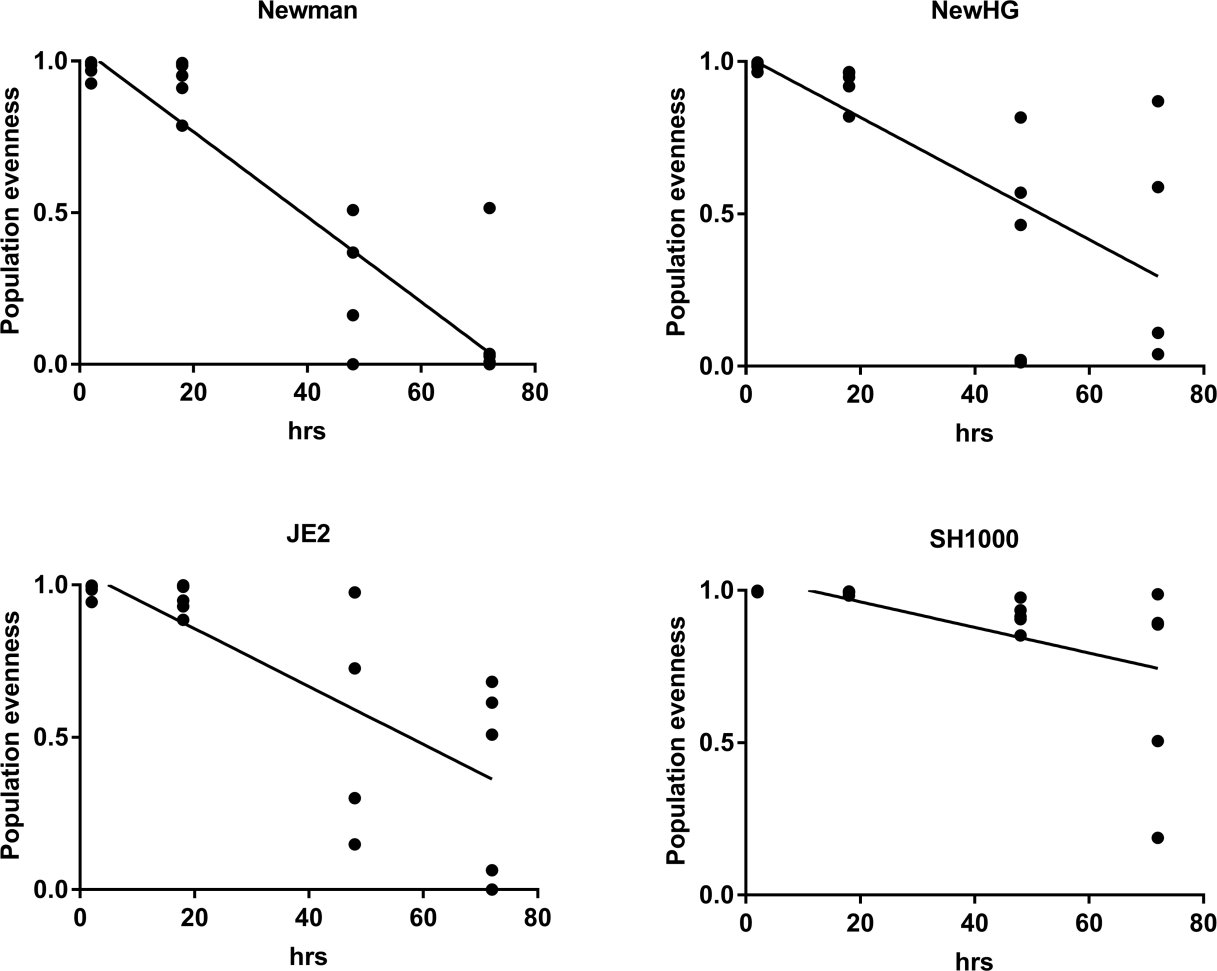
Comparison of *S*. *aureus* distribution and population evenness for the different strains in the mouse sepsis model. In mice, at all time points during infection, for all strains, the most consistently infected organ is the liver. The liver shows a statistically significant decrease in population evenness over time after infection. The dominant strain in the liver correlates significantly with the dominant strain in the other organs and can sometimes be found in all organs within a single mouse. Line: mean linear regression. Linear regression, SH1000: p = 0.0076, F = 9.018, R^2^ = 0.3338, NewHG: p = 0.0004, F = 18.82, R^2^ = 0.5254, Newman: P<0.0001, F = 73.82, R^2^ = 0.8040 and USA300: p = 0.0001, F = 24.47, R^2^ = 0.5901.

The organ most consistently infected was the liver for all strains tested (Fig 2). 2 hours after infection every liver contained bacteria (>10^5^ CFU). Between 2 and 18 hours there is a drop in the CFU and after this a great variation in liver colonisation became apparent (from clearance to 10^8^). Over time for all strains the liver showed a significant decrease in population evenness, unlike the kidneys (Fig 3, linear regression, SH1000: p = 0.0076, F = 9.018, R^2^ = 0.3338, NewHG: p = 0.0004, F = 18.82, R^2^ = 0.5254, Newman: P<0.0001, F = 73.82, R^2^ = 0.8040 and USA300: p = 0.0001, F = 24.47, R^2^ = 0.5901). The liver is the only organ that had a consistent increase in clonality over time. Thus population dynamics in the liver begins with the capture and subsequent clearance of the initial inoculum (which results in mixed populations occurring initially) and then proceeds to potentially be replaced by clonal expansion. Whether clonal expansion occurs is a stochastic process likely founded from an individual bacterium that has escaped the initial clearance of the inoculum. We have previously determined that individual abscesses are typically founded by low numbers of *S*. *aureus* (frequently a single individual) [15,18].

#### Kidneys

It is well established that within the sepsis model kidney abscesses are a key outcome [23]. However they are extremely variable both in terms of occurrence and CFU within treatment groups and between strains (lower levels of kidney infection are seen in SH1000 and Newman but greater coverage is seen in USA 300 and New HG). This indicates that the kidney is likely a key site for abscess development but the *S*. *aureus* that initiated infection in this organ have metastasised from (an)other infection site(s). This occurs after the immune bottleneck, for such variation to occur. This is further intimated to by clonality in the kidneys sampled at the various time points during the experiments not fundamentally altering (unlike in the liver). The variation in how many kidneys are infected and also how frequently one kidney is infected but the other is not further points to the population bottleneck being elsewhere. Over time in all strains the kidneys showed no overall significant change in clonality (linear regression, SH1000: p = 0.5656, NewHG: p = 0.5125, Newman: p = 0.6009 and USA300: p = 0.0587). As the organs are already consistently clonal from the earliest time points this indicates that the bottleneck occurred previously to the infection of the kidneys. The population bottlenecks are therefore found elsewhere.

#### Spleen

With strains SH1000, New HG and USA300 bacteria were found in the spleen sporadically throughout the course of the study. Whereas with Newman, significant, mixed populations were found at 2 hours and 18 hours post infection (10^2^−10^5^). As we have previously noted [13] a proportion of the original inoculum is captured from the blood by the spleen.

#### Heart & lungs

For all strains heart or lung colonisation was sporadic and CFU greater than 10^3^ were rare. However, if colonised these organs were likely clonal in that individual strains predominated.

#### Overall within host population dynamics

The above data allows a hypothesis as to the spatial dynamics within sepsis to be established. *S*. *aureus* loads found in organs apart from the kidneys and liver are low and sporadic indicating that they are likely not major contributors to the dynamics of infection. Population evenness observations in the liver and kidneys predict that the major population bottleneck for *S*. *aureus* is potentially within the liver and other organs become infected from there. In some mice, the most common marked variant in the liver matched the most common variant found in other organs and on occasion the same variant or pair of variants was found in every organ within a mouse (e.g. mouse 17 in Fig 2). There is a significant correlation between a dominant strain(s) in the liver (>50% population) and strain(s) in other organs in the same mouse (chi-squared, NewHG: p = 0.0455, USA300: p = 0.0039 and SH1000: p = 0.0034, Newman (insufficient data)). Conversely, this did not occur in the correlation between the dominant strains in the kidneys and the other organs (chi-squared, NewHG: p = 0.4450, USA300: p = 0.4028 and SH1000: p = 0.2526). Thus the liver is the source of dissemination within the host; this phenomenon would not have been detected by observing the kidneys alone, although this is a common practice (likely due to abscesses being very obvious in the kidneys) [15].

### The cellular basis for within host bottlenecks

As the liver is likely the source of within host dissemination, how is this mediated? Previously we have postulated that phagocytes form the focus for the population bottleneck and it has been demonstrated that macrophages and neutrophils are required for the control of *S*. *aureus* infections in the mouse and zebrafish models [17,28,29]. In particular, it has been shown that after iv injection, *S*. *aureus* is initially phagocytosed by Kupffer cells (liver macrophages) [30]. Neutrophils have also been demonstrated to act a potential “Trojan horses” carrying viable bacteria within themselves [5].

**Fig 4:**
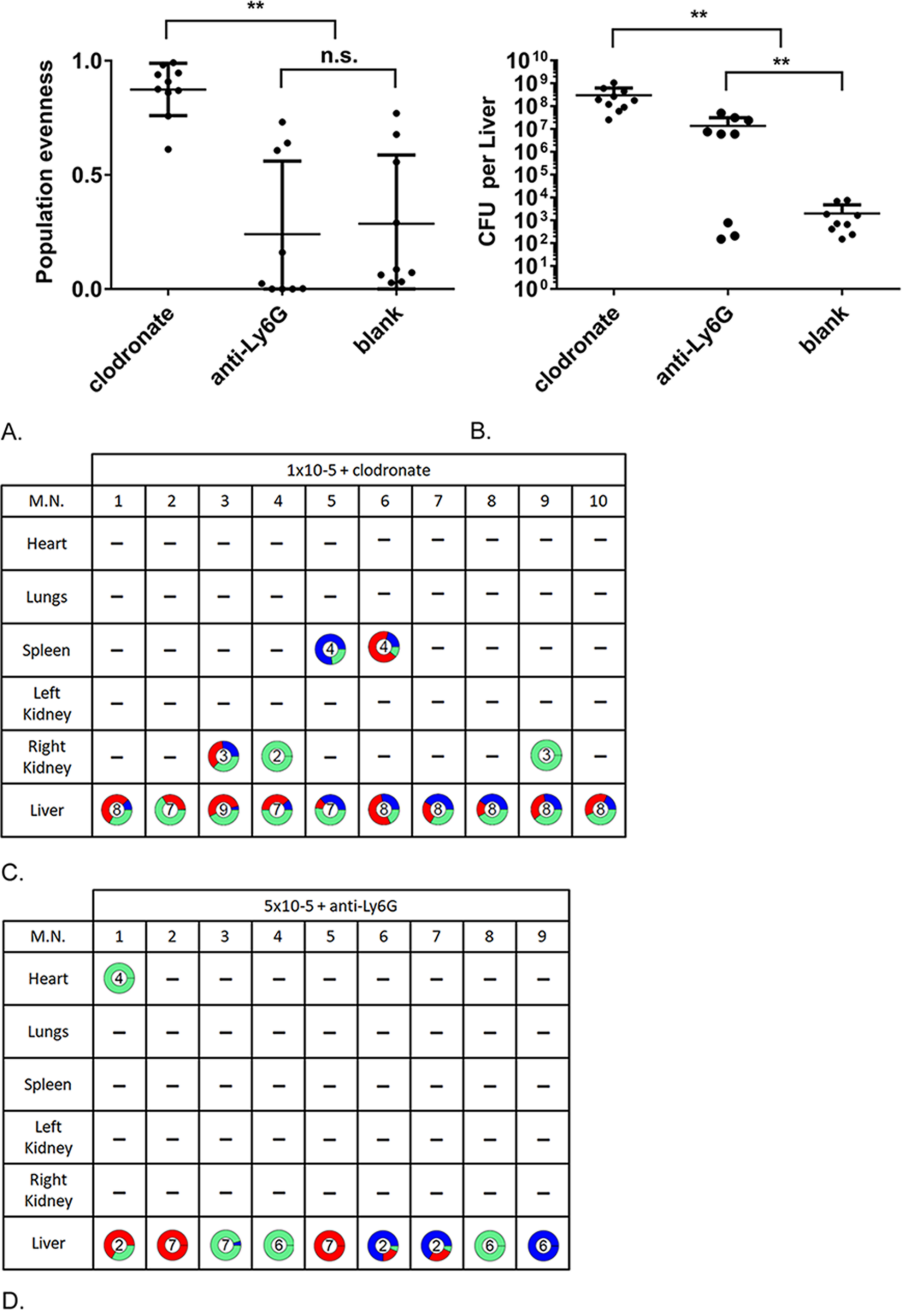
Effect of neutrophil or macrophage depletion on population dynamics during infection. Mice were treated with anti-Ly6G antibodies (neutrophil depletion) or clodronate liposomes (macrophage depletion). The bacteria were found primarily in the liver. (A) The population evenness of the bacteria in individual livers. (B) The CFU in individual livers. Error bars: mean ± SD. (C) The proportions of each strain in the various organs in macrophage depleted (clodronate treated) mice. (D) The proportions of each strain in the various organs in neutrophil depleted (anti-Ly6G treated) mice. All mice were sacrificed 3 days post infection. M.N: Mouse Number.

Macrophages were depleted using clodronate vesicles [31] and, as expected, mice are consequently more susceptible to *S*. *aureus* infection and so the dose was reduced to 1x10^5^ CFU of 1:1:1 labelled NewHG. Treated mice had a significantly higher bacterial burden in the liver compared to the control vesicle treated subjects (ANOVA, p<0.0001 Multiple comparisons show that the clodronate treated is significantly different from the blank controls, Fig 4). An additional replicate produced similar results (S5 Fig). Occasionally other organs, including the kidneys, were colonised. Interestingly, population evenness was significantly increased compared to the control (Kruskal-Wallis, p = 0.0002, Multiple comparisons show that the clodronate treated group is significantly different from the blank controls). This increased population evenness is due to the formation of multiple small abscesses visible on the liver rather than a single clone emerging.

Similarly, to macrophage depletion, neutropenia (generated using anti-Ly6G antibodies) resulted in greatly increased susceptibility to *S*. *aureus* infection with an inoculum of 5x10^5^ CFU being used (Fig 4). Neutropenic mice demonstrated detectable bacteria almost exclusively in the liver and in contrast to macrophage depletion these populations showed no difference in population evenness compared to the control, indicating clonal expansion (Kruskal-Wallis, p = 0.0002, Multiple comparisons show that the anti-Ly6G treated group is significantly different from the clodronate groups but not the blank controls). The infected livers appeared pale with no observable surface abscesses. We initially tried a lower dose of 1x10^5^ CFU, although there was neutrophil depletion, the resulting CFUs were no greater than the controls (again only the livers contained *S*. *aureus* by day 3 post infection) so we increased the dose used to the 5x10^5^ CFU results presented (additional results are shown in S5 Fig). We also depleted the neutrophils using cyclophosphamide and an inoculum of 1x10^5^ CFU. This again showed pale livers, increased CFU in the livers and mostly clonal in contrast to the macrophage depletion study (S5 Fig) and supporting our anti-Ly6G results (Liver CFU ANOVA, p<0.0001, Multiple comparisons show that the cyclophosphamide treated group is significantly different from both the blank controls and clodronate). However, the depletion of neutrophils was not as complete (cyclophosphamide is generally toxic and although it depletes neutrophils in particular it also affects lymphocytes, monocytes and other fast growing cells) so the anti-Ly6G mediated depletion was preferred for demonstrating the effect of neutrophils.

Whilst both macrophages and neutrophils are required for host defence compared to the control and their loss results in greater bacterial loads (ANOVA, P<0.0001 Multiple comparisons show that the clodronate and anti-Ly6G treated groups are significantly different from the blank controls) the macrophages, and not neutrophils, appear to be the basis for clonal expansion as their depletion results in increased population evenness. Macrophage depletion also results in greater bacterial loads than neutrophil loads when the same amount of *S*. *aureus* is administered, further indicating that macrophages are the likely bottleneck. Conversely, neutrophils appear to be involved in the seeding of organs from an already derived clonal population within the liver.

### Within organ spatial population dynamics

**Fig 5:**
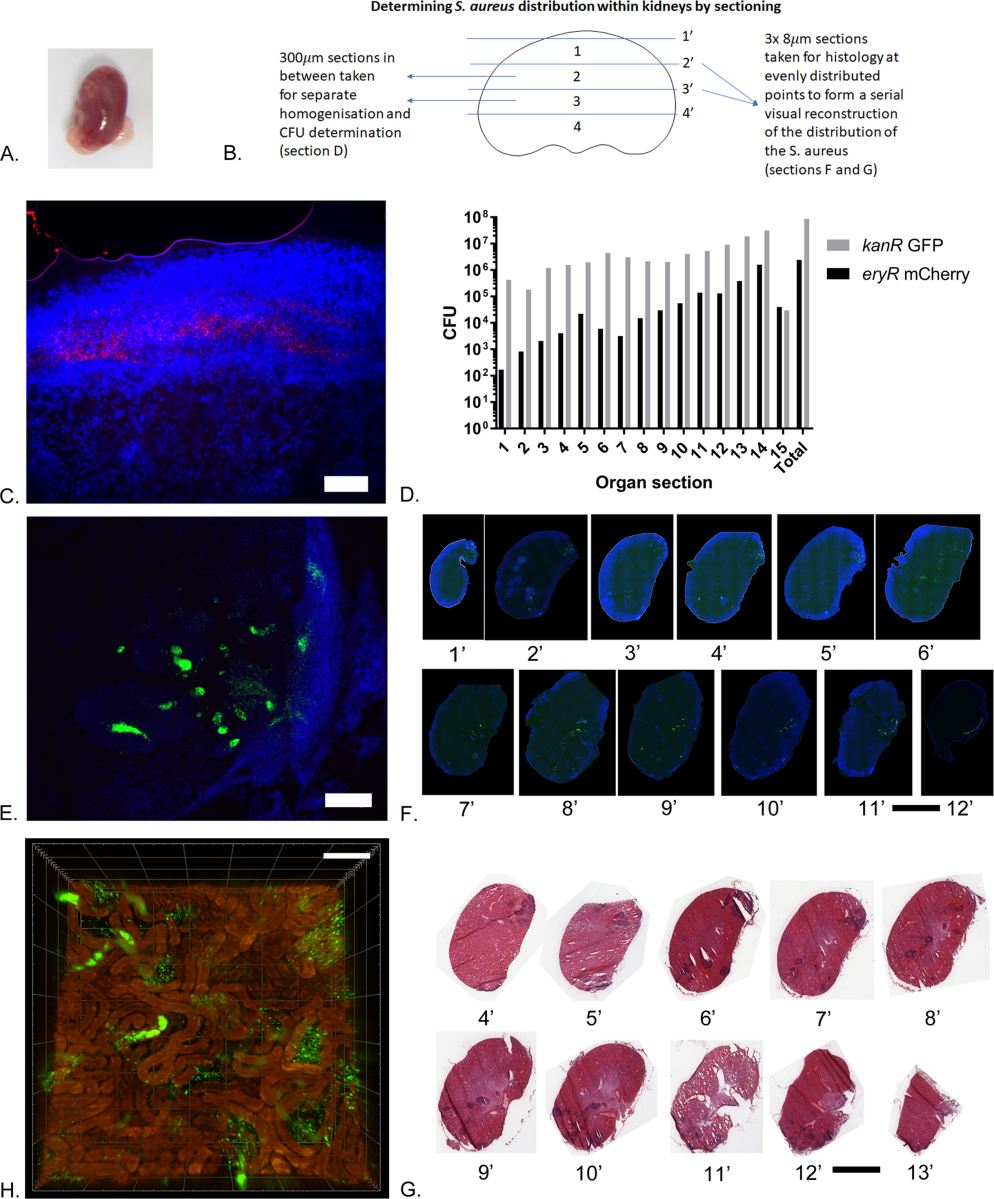
The dynamics of *Staphylococcus aureus* abscesses within a kidney organ. (A) The left kidney after dissection and before sectioning showing the multilobed abscesses throughout the kidney. (B) Schematic showing how a kidney was sectioned in order to determine the distribution of S. aureus throughout the organ both through histology (Sections F and G) and CFU determination (Section D). (C) Representative image of an *S*. *aureus* mCherry abscess. Scale bar: 100μm (D) CFU derived from homogenised sections show that the *S*. *aureus* is spatially segregated within infected organs and is unevenly distributed. (E) Representative image of *S*. *aureus* GFP abscesses. mCherry and GFP *S*. *aureus* are very infrequently found together. Scale bar: 200μm. (F) Fluorescent images showing the GFP tagged *S*. *aureus* in histology sections corresponding to the CFU sections. Scale bar: 3mm. (G) The corresponding H and E stained sections showing the abscesses. Scale bar: 3mm. (H) Summary lightsheet microscopy image of a cleared kidney abscess caused by *S*. *aureus* GFP. Scale bar: 100μm.

Kidney abscesses in the sepsis model are largely clonal in that they are derived from an individual founding cell [13,18]. However, outwardly the kidneys can show a multi-lobed abscess structure (Fig 5A). Two methods of within-organ analysis were used, both using a mixed population of differentially labelled bacteria to allow clonality to be determined *post hoc*. Firstly mice were injected with a 1:1 ratio of 1x10^7^ CFU NewHG GFP KanR/NewHG mCherry EryR, and at 5 days post infection (the time by which kidney abscesses had developed), the mice were sacrificed. Infected kidneys were serially and sequentially sectioned for CFU determination, histology and microscopy analysis of labelled bacterial populations (Fig 5B). This allowed a reconstruction of the 3D abscess architecture. Sample organs were sectioned every 300μm and 3x8μm sections of tissue were stained with either DAPI or Hematoxylin and Eosin (Fig 5F and 5G). The 300μm of tissue in between these sections was homogenised and plated for CFU determination (Fig 5D). Secondly, an optical clearing technique was employed to reveal the *in situ* distribution of fluorescently labelled bacteria within the organ (Fig 5H and S1 Video). Here lightsheet microscopy allowed a 3D rendering of the bacterial distribution to be determined [32,33].

Overall the histological sectioning revealed that there was an uneven distribution of bacteria within organs, with distinct foci that correlated with both CFU and visualisation of fluorescent bacteria (Fig 5). The foci of infection consist of solid aggregates of *S*. *aureus* or scattered individuals that co-localise with areas of neutrophil infiltration (S7 Fig). Where mature abscesses were present they almost exclusively consisted of one fluorescent variant (Fig 5C and 5D). However, this technique resulted in the destruction of the tissue and we used new techniques to confirm the distribution of *S*. *aureus* in the kidneys. We used lightsheet microscopy combined with the optical clearing of the organs, which maintains the fluorescence of the bacteria whilst making an abscess observable in 3D. Organ clearing and lightsheet microscopy has been used in other systems to great effect due to preserving structures in situ and we anticipate this technique will be of great use in *S*. *aureus* research. The lightsheet microscopy additionally showed (beyond the details revealed by histology) that within abscesses the solid aggregates in the kidneys were following the structure of kidney tubules in the cortex whilst the neutrophil infiltration sites were outside the tubules (Fig 5H and S1 Video). This would not have been demonstrated by histology alone. Based on these observations it seems likely the *S*. *aureus* is trapped in the tubules, grows into solid aggregates and then some can subsequently escape into the regions between the tubules where neutrophils engage the bacteria. Even in infected organs that contain abscesses that overall are formed from more than one marked strain, there is a clear differential distribution of clones suggesting that pervasive abscesses which have multiple foci are seeded from a single bacterium which has divided and spread to form extended foci of infection in the surrounding tissue.

### Population dynamics in the survival model

**Fig 6:**
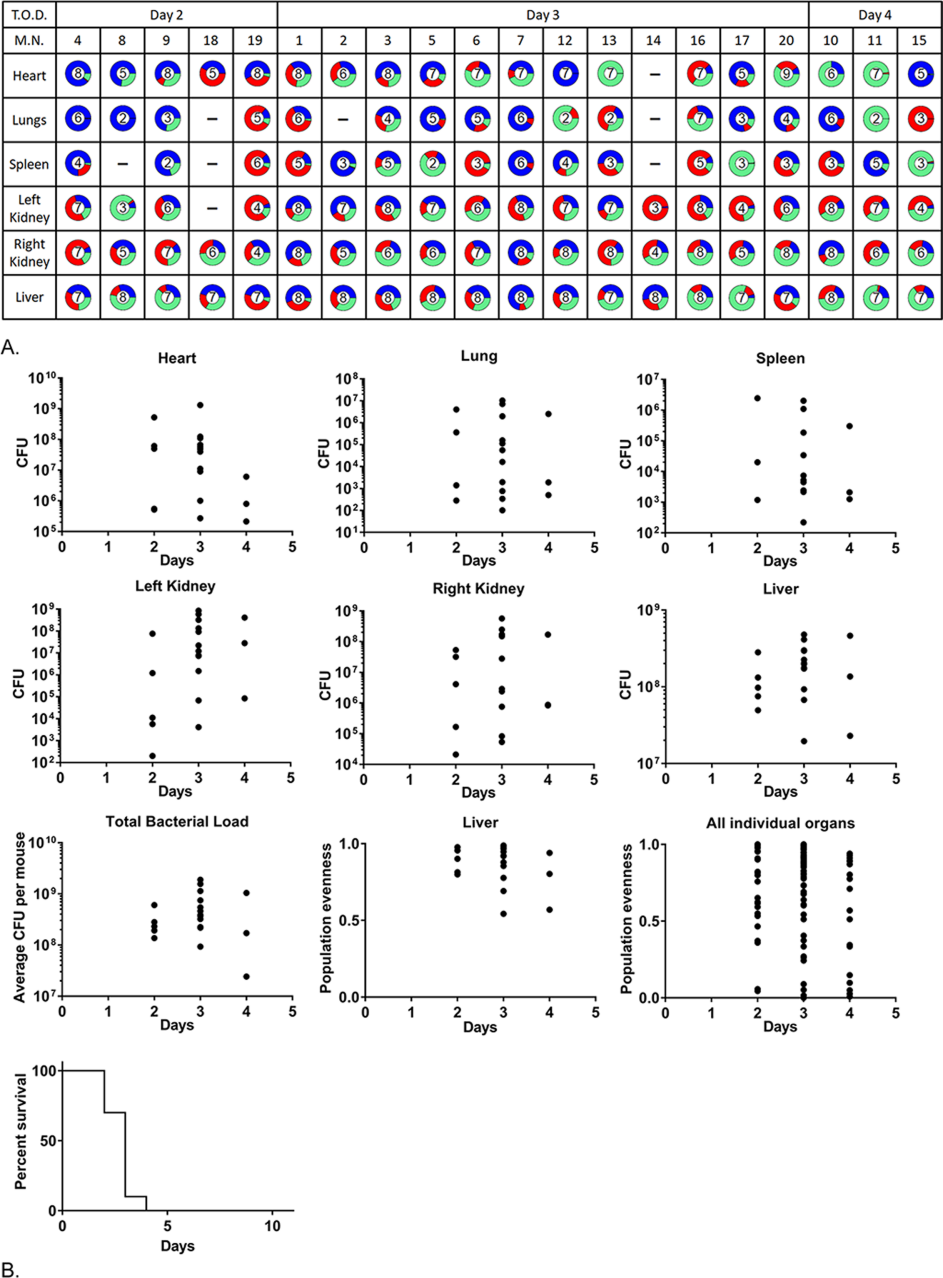
*S*. *aureus* distribution at different time points during the mouse survival challenge model. Mice were infected with a 1:1:1 mixture of 3 resistance marker tagged NewHG variants and 5 mice sacrificed as they reached the severity limits. (A) The proportions of each strain at each time point in the different organs in each mouse. The number in each represents the log amount of bacteria (e.g. 10^−6^ CFU = 6). T.O.D: Time of Death, M.N: Mouse Number. (B) The CFU load at each time point for the organs and total CFU as well as the survival curve. On each day the following numbers of mice were sacrificed due to reaching severity limits: Day 2:5, Day 3:12, Day 4:3. On Day 4 for the right kidney, 2 of the data points are overlapping.

**Fig 7:**
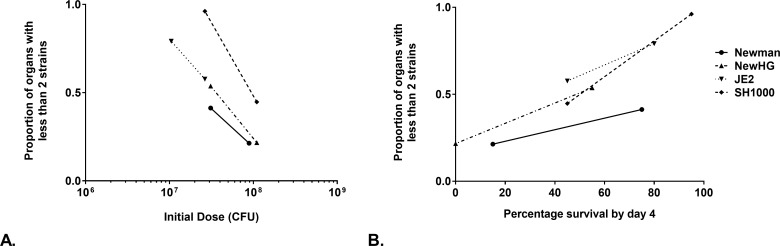
Correlation between initial inoculum, final organ clonality and survival in the mouse survival model. There is a statistically significant correlation between increasing inocula, decreasing organ clonality and decreasing survival. (A) The correlation between starting dose and final organ clonality. (B) The correlation between the proportion of mice that need culling by day 4 and organ clonality.

The mouse survival model has been used widely to test the efficacy of novel treatments, prophylaxis and the role of bacterial and host factors in disease [21,23,34–36]. It is technically similar to the mouse sepsis model but primarily differs in that an increased dose is given leading to mortality as a primary output. Strains NewHG, Newman, SH1000 and USA300 were compared using the survival model. For all strains a high inoculum was used (around 1x10^8^ CFU except USA300 which is similarly lethal at a lower dose of 3x10^7^ CFU), made up of equal proportions of the 3 antibiotic resistant marked variants in each case (Figs 6 and S3). Infection became systemic in every subject, with bacteria across multiple organs, and typically resulted in mouse cull within 2–3 days. Clonality (low population evenness) was much rarer at these high doses in all organs, in all subjects, and across all 4 bacterial strains (Fig 6). It was also notable that the hearts, lungs and spleens now had high numbers of *S*. *aureus*. To determine whether the sepsis and survival model were ends of a spectrum or independent of each other, intermediate doses of the strains were administered to delay, and reduce, the number of subjects reaching the morbidity endpoint (for all strains around 3x10^7^ CFU except USA300 which is similarly lethal at a lower dose of 1x10^7^ CFU). This resulted in a lower rate of mortality (S4 Fig), with those subjects culled up to and including day 5 having more systemic infections, whereas after this time only the liver and kidneys had bacterial loads as the infection resolves. Lower dose survival treatments had much greater levels of clonality than the equivalent higher dose survival treatment. Both kidneys are more frequently concurrently infected as well. Overall there is a significant correlation between increasing initial dose of *S*. *aureus* and the decreasing occurrence of clonality (linear regression, p = 0.0386, F = 6.959, R^2^ = 0.5370, Fig 7). There is a significant correlation between increasing clonality and increased survival by day 4 (linear regression, p=0.0049, F=18.82,R^2^=0.7583, Fig 7). There is also a significant correlation between increasing initial dose of *S*. *aureus* and decreasing survival (linear regression, p = 0.0196, F = 9.982, R^2^ = 0.6246). The order of increasing virulence in the survival model was SH1000, Newman, NewHG and USA300 consistent with previous reports [37–39].

## Discussion

As an opportunistic pathogen, *S*. *aureus* is able to cause a wide range of human diseases from the superficial to potentially life threatening. There are multiple animal models of *S*. *aureus* infection that all aim to recapitulate those events that shape the interaction between the pathogen and the human host. However, increasingly there is evidence that many of the virulence determinants are human specific and thus are not relevant to the widely used animal models. Also the route and mode of human infection is difficult to replicate in models. Murine models of *S*. *aureus* infection are commonplace and have been one of the main tools in developing our understanding of the disease processes. Models of sepsis (bacteraemia) are well-established and are characterised by kidney abscesses as a primary outcome [21,23,24]. Establishment of sepsis requires a high inoculum as is apparent in other murine models. This has been suggested to be due to a bolus of bacteria being required to initiate disease [13,15]. Recently we have shown that, in fact, there is a population bottleneck whereby likely single bacteria found kidney abscesses [13,18]. This therefore requires an explanation as to what the series of events that precedes abscess formation are, and the mechanism(s) involved. The individual bacteria that found lesions occur randomly from the population in that they do not have a genetic advantage [13,18]. Thus the initiation of abscesses is a stochastic event that is enhanced by a large inoculum. Here we aimed to investigate within-host population dynamics and determine the cellular basis of bottlenecks in *S*. *aureus* infection models.

**Fig 8:**
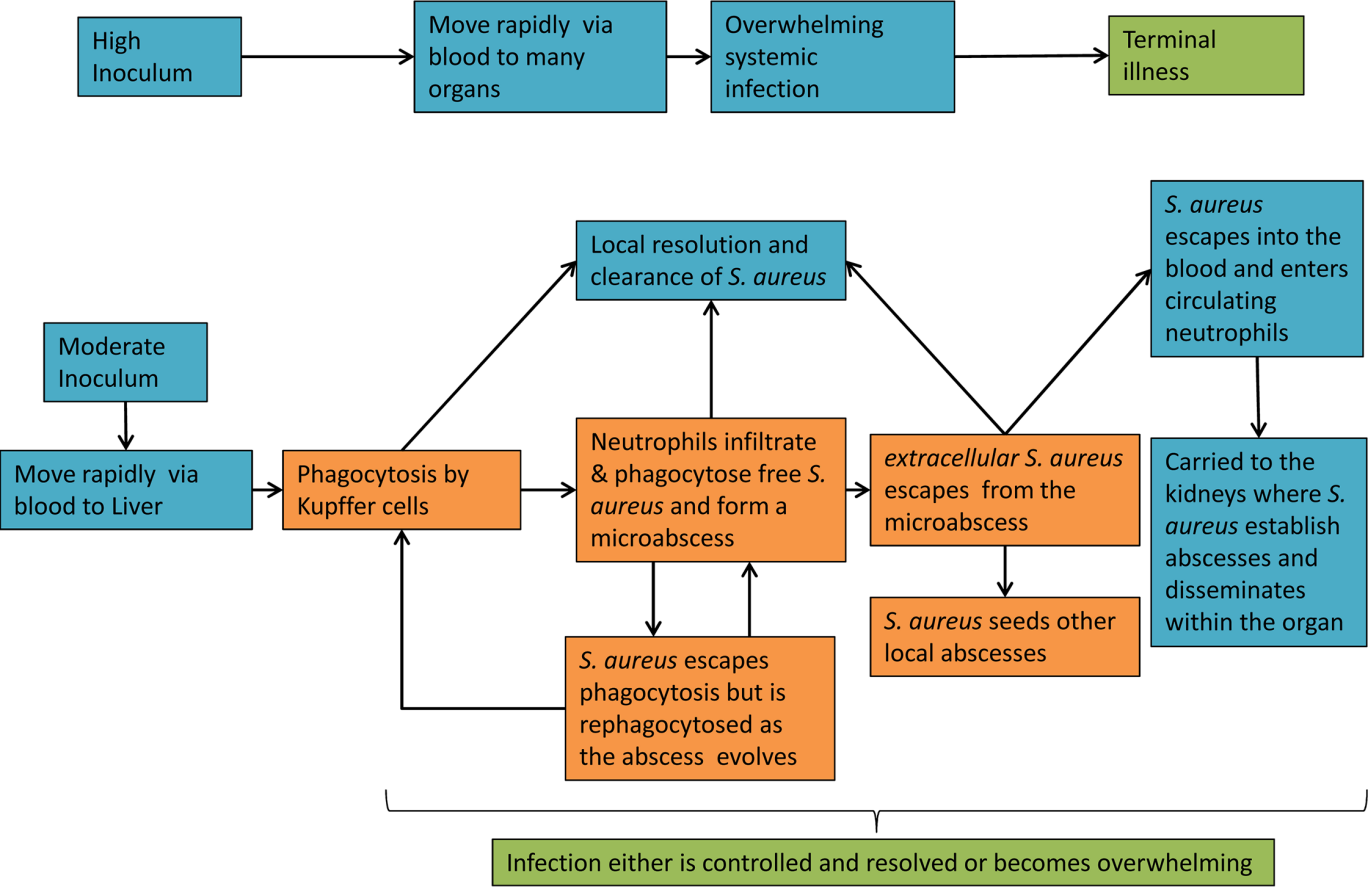
A model of the internal transmission of *S*. *aureus*. With a high inoculum the host is rapidly overwhelmed by a systemic infection. With a moderate inoculum a more dynamic infection occurs. Injected *S*. *aureus* travels through the vasculature. Bacteria are then phagocytosed by the Kupffer cells (liver macrophages), with resulting containment and killing. However a few *S*. *aureus* survive and are able to form viable extracellular microlesions. Local neutrophils are recruited to aid in their control. As the abscess develops some *S*. *aureus* can likely escape into the blood. *S*. *aureus* can then seed the kidneys that can then go on to form large clonal abscesses. If both macrophages and neutrophils are unsuccessful in clearing the infection in the liver then clonal abscesses can also form there.

The temporal and spatial dynamics of sepsis in zebrafish and murine models have been determined using sets of marked strains in a range of backgrounds. This enabled a model for the dynamics of infection in the mouse to be established (Fig 8). We found for all strains that the liver was the key destination organ for the initial mixed population inoculum. Previously *S*. *aureus* has been shown to be phagocytosed by Kupffer cells in the liver and these form a primary immunological defence (2, 22). Subsequently expansion of individual clones occurs in the liver or the bacterial load is cleared. Kupffer cells are effective agents for the control of *S*. *aureus* but if this immunological bottleneck fails then bacteria are able to grow to form abscesses. Depletion of Kupffer cells results in greatly increased host susceptibility to *S*. *aureus* infection manifested by abscesses mostly in the livers, but also in the kidneys. Kidney abscesses develop during the infection but these organs are not an initial colonisation site. Interestingly kidneys do not have mixed populations that then become clonal but rather this happens at abscess initiation suggesting that they are founded by single organisms or that the founders were already clonal. Given the complex population dynamics in the liver we suggest that it is the transfer of *S*. *aureus* from the liver that gives rise to kidney colonisation. But how therefore do the bacteria traffic between organs? A clue to this comes from the generation of neutropenia within the mouse. Interestingly loss of neutrophils results in no loss of clonality within the liver. Thus the neutrophils are not the primary bottleneck within the liver. However, subsequent abscess formation was abrogated within the kidneys. The importance of neutrophils for dissemination correlates with other research that shows that *S*. *aureus* can live intracellularly within phagosomes and be transported by them in the blood, forming a mobile reservoir to infect other organs [5,40,41]. Also, treatment with antibiotics that do not penetrate the neutrophil phagosome do not prevent internal dissemination of *S*. *aureus* [30,42]. However, conventionally neutrophils that mature and enter infected tissue do not re-enter the blood stream [28,43]. The most parsimonious explanation is that neutrophils that are already circulating in the blood (whose population greatly increases in response to infection) take up *S*. *aureus* that escape into the blood stream from the lesions in the liver. This could be due to abscesses/microlesions shedding *S*. *aureus* into the blood stream, as is known to occur in clinical infections [44].

Kidney CFU alone is used as a readout in many studies of *S*. *aureus* disease, for which one might express caution as if bacteria have passed through the liver bottleneck then they can clonally expand and so the subtleties of *S aureus* infection prior to this point will be lost. Liver CFU provides a useful adjunct to kidney numbers. It might seem surprising that a kidney with many apparent surface abscesses could in fact be seeded by an individual founder. It is also important to note that it is unlikely that apparently clonal organs are founded by multiple bacteria of the same type as left and right kidneys are often clonal but with different clones. In order to determine how a single organism could establish a disseminated abscess throughout a kidney we began to map the 3D architecture of the abscess. We developed two methods to address this conundrum. Serial organ sectioning revealed foci of infection with the characteristic abscess structure of infiltration of phagocytes but these extended throughout the kidney forming a seemingly linked series of lesions. Organ clearing is a new approach to study infecting organisms within host tissue and here it revealed a pervasive spread of *S*. *aureus* as solid aggregates within cortex tubules and as diffuse groupings with infiltration of phagocytes outside these tubules [33,45].

The scheme presented in Fig 8 suggests the presence of two immunological bottlenecks within the murine sepsis model. Firstly, at the level of Kupffer cells in the liver and then at the neutrophils when disseminating from the liver. Zebrafish embryos do not have Kupffer cells at the time of infection. In the zebrafish the population bottleneck occurs in the phagocytes but is mainly believed to be due to the neutrophils[18]. Our mouse models therefore apparently diverge from the zebrafish model on the relative importance of the two major classes of professional phagocytes. This study provides data that phagocytes present a key breakpoint during infection in that these cells are absolutely required for host resistance but also permit proliferation as “Trojan Horses” as has been previously proposed [40]. The question arises however as to whether this hypothesis translates into a human infection both in terms of the immune system and the occurrence of population bottlenecks. The murine sepsis model shares several characteristics that are known to occur within human infections such as how *S*. *aureus* can disseminate in the blood stream to different organs from an initial infection site and the formation of localised abscesses in separate organs.

It is known that professional phagocytes are key to controlling *S*. *aureus* infections but that *S*. *aureus* can also hide intracellularly [5,28]. What we have found correlates with human infections. In our model both neutrophils and macrophages are important for controlling *S*. *aureus* infection and the same is true in humans as shown by various genetic disorders in these can result in increased *S*. *aureus* infections [46]. This is however more commonly associated with neutrophil disorders (which may reflect how macrophages are generally less dispensable as they are required for tissue development and homeostasis).

One particular point is that our model shows that the liver is the site of the primary bottleneck. Generally, in humans, the liver is not a site particularly associated with *S*. *aureus* infection (compared to soft tissue infections or deep infections such as osteomyelitis) so it would seem unlikely it would have the same role in humans. However, patients with chronic granulomatous disease (lacking the respiratory burst part of phagocytosis) have a particularly high risk of staphylococcal liver abscesses [46,47]. This implies that in normal people *S*. *aureus* can be cleared from the liver as a normal process, which would be by the action of Kupffer cells, the resident professional phagocytes. Our results suggest that the killing activity of macrophages on *S*. *aureus* should be worthy of further study but also the ability of neutrophils to transfer *S*. *aureus* between organs should also be of great interest as has been shown by other methods [5,30].

Genome studies have shown that there are repeated genetic population bottlenecks and that these occur both during transmission and the shift from colonisation to disease [48–50]. In human infections, population bottlenecks would be interacting with the inherent variation of the naturally colonising *S*. *aureus* population as infections tend to be derived from the previous resident *S*. *aureus* [48]. The existence of stochastic niches and resource limitation (here access to host nutrients) according to ecological studies are intrinsically supposed to maintain increased population diversity particularly if they are better at competing for different resources [51]. Population bottlenecks, as the host is infected, would form stochastic niches and the exact site would exert different selection pressures. They could therefore work to maintain population diversity and increased strain divergence as the population bottleneck would randomly exclude different subpopulations upon each infection. This may be reflected in the diversity of virulence factors that different strains of *S*. *aureus* possess and the maintenance of different clones of *S*. *aureus* [52]. Population bottlenecks would also favour variants that are better at getting through it rather than selecting for mutants fitter after subsequent diversification. This concurs with the diversity seen in Young during colonisation and the more limited subsequent diversification [48].

A population bottleneck results in a notable distribution of the observable population. There are members of the administered dose that pass through and come to dominate the population and then there are those that are excluded, which ultimately means there tends to be two groups of data: mixed populations that have not yet passed through the bottleneck and populations that have become, or are rapidly becoming, clonal (as one strain is expanding relative to the others). This can be seen in our data as organs with a mixed population with all 3 strains present and organs with clonal populations. It is reasonable to make assessments based on the proportion of a population that was mixed or clonal as we have done but it would be much harder to assess if one population was becoming clonal more quickly than another (e.g. if one strain or another was better at expanding out of the macrophage bottleneck).

Population bottlenecks during infection represent key breakpoints for interventions. Despite apparent success in animal models, vaccine development for *S*. *aureus* has not translated into successful human trials [10,53]. Active vaccination is by its nature prophylaxis and thus the target should be to prevent the ability of *S*. *aureus* to pass through bottlenecks and proliferate. The knowledge that intracellular organisms form this nexus within phagocytes provides a key parameter from which to design future vaccine formulations. Recent evidence highlighting the targeting of intraphagocytic *S*. *aureus* supports such an approach [42]. This is consistent with what has been found previously, in that *S*. *aureus* can survive within neutrophils; and treating mice with antibiotics that do not affect intracellular *S*. *aureus* does not prevent dissemination [5,30].

Our work, as well as highlighting the cellular locations of immunological bottlenecks also sheds light on the role of the infectious dose on the outcome of infection and the population dynamics therein. The survival challenge model is a standard in the field and has been used in many studies [21,24]. Measuring population dynamics within this model showed a clear diminution of population bottlenecks correlating with an increased infectious dose leading to host morbidity. High infectious doses result in systemic infections across all major organs. The relevance of this model is therefore bought into question as clearly those immunological processes that control the dynamics of infection have been overwhelmed. This observation is therefore of great importance for the design of intervention studies where one is modelling human infection and not uncontrolled bacteraemia. Using lower doses is more realistic as well as allowing population bottlenecks to occur as infectious doses of *S*. *aureus* are likely to be inherently lower in humans compared to our mouse models. In Grice et al punch biopsies were used which gave total bacterial numbers of at least 10^6^ CFU/cm^2^[54]. In a study of vascular catheters, a range of bacteria were found with numbers up to 10^7^ CFU [55]. In a given infection it is likely that only a fraction of these would be able to disseminate from the skin or implant. This is reflected in bacteraemia where the count is typically <10 CFU per ml [56]. The number of bacteria that can transfer to infect organs is much less than >5 x 10^7^ CFU per ml murine blood if 10^8^ CFU is injected (assuming 1.5 ml of blood per 25g mouse).

The threat of antimicrobial resistance to human healthcare is real and increasing. Disease is a complex and dynamic interplay between host and a population of infectious agent. Here we have highlighted key parameters of how *S*. *aureus* disease progresses and provide a framework for determination of efficacy of new interventions.

## Materials & methods

### Ethics statement

Animal work (both mice and zebrafish) was carried out according to guidelines and legislation set out in the UK Animals (Scientific Procedures) Act 1986, under Project Licenses PPL 40/3699 and PPL 40/3574. Ethical approval was granted by the University of Sheffield Local Ethical Review Panel.

### Bacterial strains and growth conditions

*Staphylococcus aureus* strains (S1 Table) were grown using brain heart infusion (BHI) liquid or solid medium (Oxoid) at 37°C, supplemented with the following antibiotics where appropriate: kanamycin 50 μg/ml, tetracycline 5 μg/ml or erythromycin 5 μg/ml plus lincomycin 25 μg/ml (Sigma-Aldrich).

### Construction of antibiotic resistant strains

*S*. *aureus* TetR, EryR and KanR strains used included those constructed previously [13]. The resistance markers were transferred into further strain backgrounds using ϕ11 transduction [57]. ϕ11 variants of suicide vector pMUTIN4 were used to integrate various antibiotic resistance cassettes downstream of the *lysA* gene in *S*. *aureus* in the original strains [13]. The genomic region surrounding *lysA* is conserved in the Newman and USA300 genomes.

### Construction of fluorescent strains

The suicide vector pKASBAR (and pKASBARkan) was used to integrate constitutive GFP and mCherry fluorescence markers with promoter Pma1M from the *E*. *faecalis* pmv158GFP and pmv158mCherry plasmids into the *S*. *aureus* lipase gene in RN4220 [58,59]. The fluorescent markers were then transferred into SH1000, Newman, NewHG and USA300 by ϕ11 transduction. This resulted in strains which were GFP KanR or mCherry TetR.

### Construction of fluorescent strains–*GFP* strains

A fragment of c.1200 bp containing the GFP encoding gene and its promoter sequence was amplified by PCR from pMV158GFP plasmid using the following primers:

EP01 cctttttttgccccgggatcCTATTTGTATAGTTCATCCATGC
EP02 ctatgaccatgattacgaattcAGCTTGATTTGATAGCATAATTTTG

The PCR fragment was cloned into pKASBARkan by Gibson assembly, with the plasmid cut by EcoRI and BamHI [58].The resulting plasmid pKASBARGFPkan was introduced into *E*. *coli* NEB5-alpha by electroporation with selection for ampicillin resistance. The correct plasmid was confirmed by sequencing and used to transform *S*. *aureus* competent cells RN4220 containing a helper plasmid pCL112Δ19 and then selected for with Kanamycin[60]. The marker was then transferred into the NewHG strain (and other backgrounds) by ϕ11 transduction.

### Construction of fluorescent strains–*mCherry* strains

A fragment of c.1200 bp containing the mCherry encoding gene and its promoter sequence was amplified by PCR from pMV158mCherry plasmid using the following primers:

PTS01 cctttttttgccccggGAGCTCGGTACCCAGCTT
PTS02 ctatgaccatgattacgTTAATGATGATGATGATGATGAGATCTTTTATATAATTC

The PCR fragment was cloned into pKASBAR by Gibson assembly with the plasmid cut by EcoRI and BamHI[58]. The resulting plasmid pKASBARmCherry was introduced into *E*. *coli* NEB5-alpha by electroporation with selection for ampicillin resistance. The correct plasmid was confirmed by sequencing and used to transform *S*. *aureus* competent cells RN4220 containing a helper plasmid pCL112Δ19 and then selected for with tetracycline [60]. The marker was then transferred into NewHG (and other backgrounds) by ϕ11 transduction. The generated strains however showed only weak tetracycline resistance and were therefore supplemented by Ery^R^ from SJF3673 strain collection (*lysA*::*ery lysA*^*+*^) by ϕ11 transduction. Transductants with supplemented Ery^R^ were verified by PCR.

### Zebrafish maintenance and microinjection

London wild-type (LWT) zebrafish embryos (bred in the MRC CDBG aquarium facilities at the University of Sheffield; see Ethics Statement) were used for all experiments and were incubated in E3 medium at 28.3°C according to standard protocols [61]. Anaesthetized embryos at 30 hours post fertilization were embedded in 3% w/v methylcellulose and injected individually using microcapillary pipettes filled with bacterial suspension of known concentration into the blood circulation, as previously described [17]. Following infection, embryos were kept individually in 100 μl E3 medium, and observed frequently up to 90 hours post infection; dead embryos removed and CFU from these embryos recorded at each time point.

### Intravenous mouse injections

6–7 week old Female BALB/c mice were purchased from Envigo (formerly Harlan (UK)) and maintained at the University of Sheffield using standard husbandry procedures. The mice were acclimatised for 1 week. The mice were then inoculated in the tail vein with 100 μl of *S*. *aureus* suspension in endotoxin-free PBS (Sigma) diluted from frozen stocks. Viable bacteria in the inoculum were plated on TSB (plus appropriate antibiotics) after serial decimal dilution to confirm the accuracy of the bacterial dose. Mice were monitored and sacrificed at various time-points according to experimental design.

### Mouse sepsis model design

All mice were injected with 1x10^7^ CFU *S*. *aureus* consisting of a 1:1:1 ratio of KanR, EryR & TetR variants of the different strains (SH1000, Newman, NewHG, USA300). 5 mice were sacrificed at the following time points post infection: 2hrs, 18hrs, 48hrs, 72hrs (end of procedure)

### Mouse survival challenge model design

The mice were injected with the following doses of *S*. *aureus* to reflect different levels of challenge that result in both low and high levels of mice that reach the severity limits. The higher dose set: SH1000: 1x10^8^ CFU, NewHG: 1x10^8^ CFU, Newman: 9x10^7^ CFU, USA300: 3x10^7^ CFU The lower dose set: SH1000: 3x10^7^ CFU, NewHG: 3x10^7^ CFU, Newman: 3x10^7^ CFU, USA300: 1x10^7^ CFU.

### Immune modulation–Macrophage depletion

Macrophages were depleted using clodronate liposomes following previously published protocols (NvR, http://www.clodronateliposomes.org/)[30,31]. The mice were injected iv with 1ml of liposomes per 100g on day 1. The mice were then injected with 1x10^5^ CFU *S*. *aureus* on day 2. Mice were sacrificed on day 5 (3 days post infection). Blank liposomes were used as a control. Macrophage depletion was confirmed in pilot studies using histology sections of the liver stained with F4/80 from Serotec AbD Serotec MCA497, followed by an anti-rat red fluorophore. The results confirming macrophage depletion are shown in S6 Fig.

### Immune modulation–Neutrophil depletion

Neutrophils were depleted using anti-Ly6G mouse antibodies following previously published protocols [62]. For antibody based neutrophil depletion Invivo anti-Ly6G mouse antibody (1A8, BioXcell) was used [62]. The mice were injected with 1.5mg/mouse (200ul per mouse) on day 1 with the mice being injected with *S*. *aureus* on day 2. Mice were sacrificed on day 5 (3 days post infection).

For cyclophosphamide depletion mice were injected intraperitoneally with 150mg/kg cyclophosphamide monohydrate (Sigma, also known as Cyclophosphamide & Cytoxan) on day 1 and 100mg/kg cyclophosphamide intraperitoneally on day 4. The mice were injected with 20mg/ml cyclophosphamide reconstituted in etox free water. The mice were then injected with *S*. *aureus* on day 5. Mice were sacrificed on day 8 (3 days post infection).

Neutrophil depletion was confirmed using flow cytometry. 100μl of blood was collected via tail bleeding at the same time as *S*. *aureus* was injected and 100μl was collected at the end of the experiment via terminal anaesthesia and heart puncture. These blood samples were each mixed with 20μl of Heparin. The blood samples were stained with APC Rat Anti-Mouse Ly6G antibody (BD biosciences, cat. No: 560599) according to the BD bioscience protocol for Immunofluorescent Staining of Mouse and Rat Leukocytes and Fixation with fixation buffer (cat. No: 554655). (http://www.bdbiosciences.com/eu/resources/s/mouseratleukocytes). The samples were then processed using the BD LSRII flow cytometer. The results confirming neutrophil depletion are shown in S6 Fig.

### Determination of in vivo bacterial load

In order to recover bacteria from host tissues, whole zebrafish embryos or mouse organs were individually homogenized in a suitable volume of PBS using the PreCellys 24-Dual (Peqlab) [13]. Homogenates were serially diluted in PBS and plated on TSB (*S*. *aureus*) or TSB supplemented with appropriate antibiotics (tetracycline, erythromycin or kanamycin) to determine bacterial numbers. The limit of detection was defined as <100 CFU as defined previously and these results were treated as 0 CFU [13].

### Tissue sectioning and visualisation of fluorescent strains

Mice were injected with a 1:1 ratio of 1x10^7^ CFU NewHG GFP KanR/NewHG mCherry EryR, and at 5 days post infection the mice were sacrificed, organs with notable abscesses were sectioned and histology performed. The selected organs were sectioned evenly throughout. Every 300μm, 3 sections (8μm each) of tissue were stained with either DAPI, Hematoxylin and Eosin, or Gram stain. The 300μm sections in between were homogenised separately and plated out for bacterial enumeration as described above. DAPI stained tissue sections were analysed using a Nikon Dual Cam fluorescent microscope whilst the Hematoxylin and Eosin or Gram stained slides were analysed using an Aperio digital microscope slide scanner. Other organs were optically cleared using the ScaleS protocol and were visualised using a Zeiss Z1 lightsheet microscope [33].

### Statistical analysis

Survival experiments were evaluated using the Kaplan-Meier method. Comparisons between curves were performed using the log rank test. For comparisons between CFU groups, a two-tailed, unpaired ANOVA was used. For comparisons of strain ratios where 3 strains were tested (e.g. TetR, EryR and KanR), species evenness was calculated per sample (Species evenness is Shannon’s diversity index H divided by the natural logarithm of species richness ln(S)) and then compared using a (non-parametric) Kruskal-Wallis test. Bartlett’s test for equal variance was used to test if the mixed strains were equally fit (population spread should be equal). For comparing correlations linear regression was used and the mean was presented on corresponding graphs. All statistical analysis was performed using Prism version 6.0 (GraphPad) and statistical significance was assumed at p<0.05. Error bars indicate mean ± One Standard Deviation.

## Supporting information

S1 Fig Numbers of each strain in the zebrafish and mouse sepsis model for demonstration of equal fitness. The combined CFU from each strain for each individual zebrafish in the zebrafish studies (A) and for all organs for each individual mouse is presented for the mouse sepsis studies (B). This demonstrates that in both models that none of the marked strains has a survival advantage over the other marked strains.
(TIF)

S2 Fig ***S*. *aureus* distribution at different time points during the mouse sepsis model for SH1000 (A), Newman (B) and USA300 (C). **Mice were infected with a 1:1:1 mixture of 3 resistance marker tagged variants for each strain. For each panel, above shows the proportions of each strain at each time point in the different organs in each mouse. The number in each represents the log amount of bacteria (e.g. 10^−6^ CFU = 6). Below shows the CFU load at each time point for the organs and total CFU. Organs with CFU counts below the limit of detection (<100CFU) are represented by open circles. Error bars: mean ± SD. 5 mice were sacrificed in each study at 2hrs, 18hrs, 48hrs and 72hrs post injection of *S*. *aureus*.
(PDF)

S3 Fig ***S*. *aureus* distribution at different time points during the mouse survival model for USA300 (A), Newman (B) and SH1000 (C).** Mice were infected with a 1:1:1 mixture of 3 resistance marker tagged variants and 5 mice sacrificed as they reached the severity limits. For each panel, above is shown the proportions of each strain at each time point in the different organs in each mouse. The number in each represents the log amount of bacteria (e.g. 10^−6^ CFU = 6). Below is shown the CFU load at each time point for the organs and total CFU as well as the survival curve. The population evenness of the liver and all the individual organs is also shown. For the USA300 study, on each day the following numbers of mice were sacrificed due to reaching severity limits: Day 2:4, Day 3:4, Day 4:3, Day 5:2, Day 6:2, Day 11: 5 (end of procedure). For the Newman study, on each day the following numbers of mice were sacrificed due to reaching severity limits: Day 2:1, Day 3:8, Day 4:8, Day 5:3. For the SH1000 study, on each day the following numbers of mice were sacrificed due to reaching severity limits: Day 1:1, Day 2:4, Day 3:3, Day 4:4, Day 5:2, Day 6: 2, Day 11: 4 (end of procedure).
(PDF)

S4 Fig ***S*. *aureus* distribution at different time points during the mouse survival model (lower dose) for NewHG (A), SH1000 (B), USA300 (C) and Newman (D).** Mice were infected with a 1:1:1 mixture of 3 resistance marker tagged variants and 5 mice sacrificed as they reached the severity limits. Here the mice were given a substantially lower dose than in the other survival studies. For each panel, above is shown the proportions of each strain at each time point in the different organs in each mouse. The number in each represents the log amount of bacteria (e.g. 10^−6^ CFU = 6). Below is shown the CFU load at each time point for the organs and total CFU as well as the survival curve. The population evenness of the liver and all the individual organs is also shown. For the NewHG study, the following numbers of mice were sacrificed due to reaching severity limits: Day 2:4, Day 3:3, Day 4:2, Day 5:3, Day 8:1, Day 9:1, Day 10:1, Day 11:5 (end of procedure). For the Newman study, the following numbers of mice were sacrificed due to reaching severity limits: Day 3:3, Day 4:2, Day 5:5, Day 6:2, Day 7:1, Day 8:3, Day 10:1, Day 11: 3 (end of procedure). For the USA300 study, on each day the following numbers of mice were sacrificed due to reaching severity limits: Day 2:2, Day 4:2, Day 11: 16 (end of procedure). For the SH1000 study, on each day the following numbers of mice were sacrificed due to reaching severity limits: Day 4:1, Day 6:1, Day 8:1, Day 11: 17 (end of procedure).
(PDF)

S5 Fig Macrophage and neutrophil depletion studies. (A) The proportions of each strain in the various organs in control mice (blank liposomes) injected with 1x10^5^ CFU (counts and diversity shown in Fig 4A and 4B). (B) The proportions of each strain in the various organs in mice injected with 1x10^5^ CFU and clodronate. In a repeat of the previous study shown in Fig 4, mixed populations occur in the liver and spread to other organs occurs. (C) The proportions of each strain in the various organs in mice injected with 1x10^5^ CFU and anti Ly-6G. Again the liver populations are largely clonal and there is no spread to other organs. However the numbers were equivalent to the blank controls so consequently the dose was increased the dose for the depletion study (Fig 4). (D) The proportions of each strain in the various organs in mice injected with 1x10^5^ CFU and cyclophosphamide. There are higher loads in the livers and clonality occurs. All mice were sacrificed 3 days post infection apart from the cyclophosphamide study where the mice were sacrificed due to hitting severity limits as follows: Day 1:2, Day 2:5, Day 3: 3 (end of procedure). (E) The CFU in individual livers. (F) The population evenness of the bacteria in individual livers. Error bars: mean ± SD.
(PDF)

S6 Fig Macrophage and neutrophil depletion controls. (A) Representative liver sections from a control mouse, (B) Representative liver sections from a mouse treated with clodronate. Both sections were stained with antibody against the macrophage marker F4/80 (Biorad, MCA497) then stained with an anti-rat red fluorescent antibody. Few if any macrophages are visible in the clodronate treated mice. Scale bars: 50μm. (C) Representative flow cytometry from a non-treated control mouse. (D) Representative flow cytometry from a mouse treated with anti-Ly6G antibody. (E) Proportional neutrophil counts in Neutrophil depleted mice against a control group of non-treated mice (n- = 3). Very few neutrophils are detectable in treated groups compared to the infected controls, although some are present in the cyclophosphamide treated group.
(PDF)

S7 Fig Neutrophils are present in abscesses in kidneys. Neutrophils are found surrounding *S*. *aureus* aggregates in abscesses as shown in serial sections. (A) One section showing a *S*. *aureus* GFP aggregate. (B) The serial section below stained with Haematoxylin and Eosin showing the neutrophils surrounding it. (C) Another section showing a mix of aggregates and diffuse groupings of *S*. *aureus* GFP. (D) The serial section below stained with Haematoxylin and Eosin showing the neutrophils surrounding these bacteria as well as fibrosis around the abscess.
(PDF)

S1 Video *S*. *aureus* distribution within an abscess. A lightsheet microscope was used to visualise a kidney abscess caused by *S*. *aureus* GFP that had been cleared with the scale S technique. The video rotates around a mature kidney abscess starting outside the kidney, the *S*. *aureus* GFP (green) is readily visible whilst the background fluorescence of the tissue has been used to render the kidney tubules observable. Within the abscess the *S*. *aureus* GFP is found both as solid aggregates within the microtubules and diffuse groupings outside the microtubules (it is likely they have been phagocytosed by neutrophils here), indicating heterogeneous behaviour of the *S*. *aureus*. This would not have been directly observable without this technique.
(MP4)

S1 Table Bacterial strains and plasmids used in this study. (PDF)
