# Supplementary figures and images for: Staphylococcus aureus infection dynamics
Source: PLoS Pathog. 2018 Jun 14;14(6):e1007112. doi: 10.1371/journal.ppat.1007112 (PMC6019756; doi:10.1371/journal.ppat.1007112)

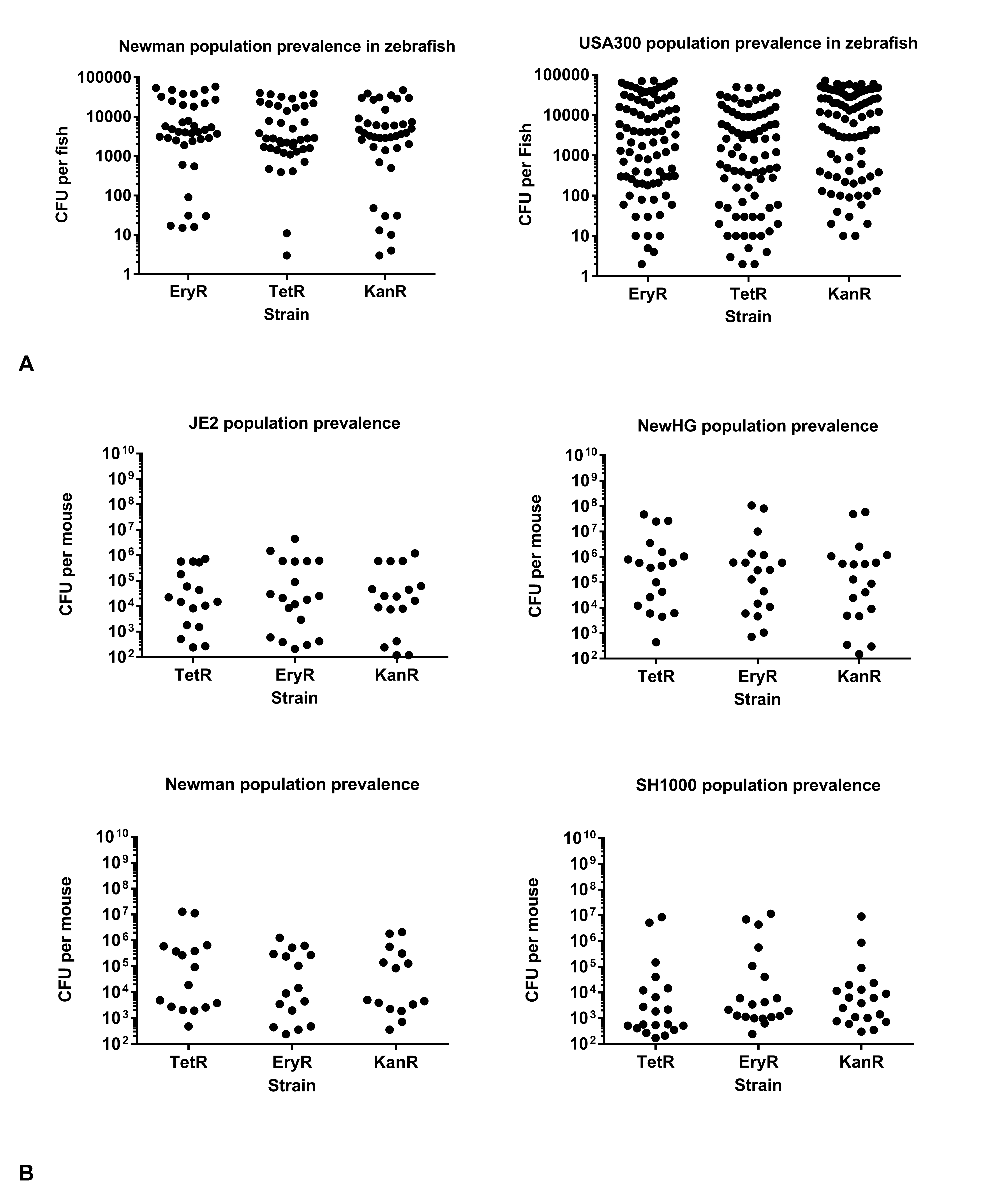

Supplement: S1 Fig — The combined CFU from each strain for each individual zebrafish in the zebrafish studies (A) and for all organs for each individual mouse is presented for the mouse sepsis studies (B). This demonstrates that in both models that none of the marked strains has a survival advantage over the other marked strains. (TIF) [file ppat.1007112.s001.tif]

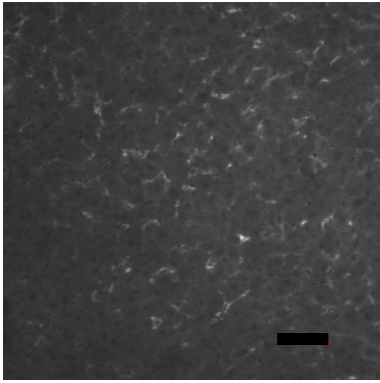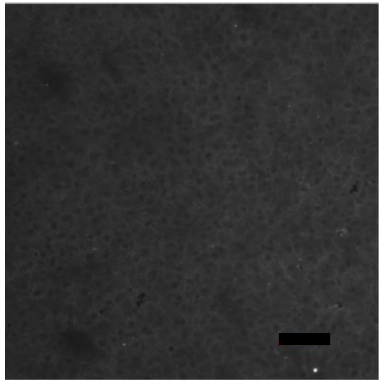

A.

B.

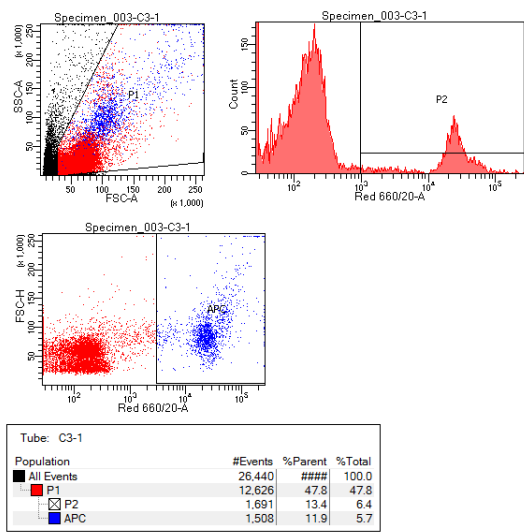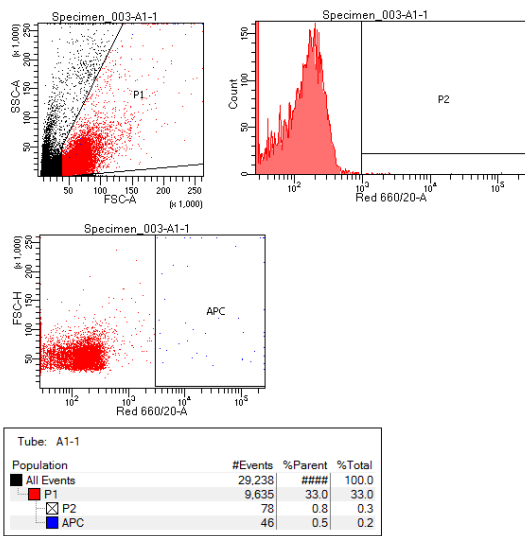

C.

D.

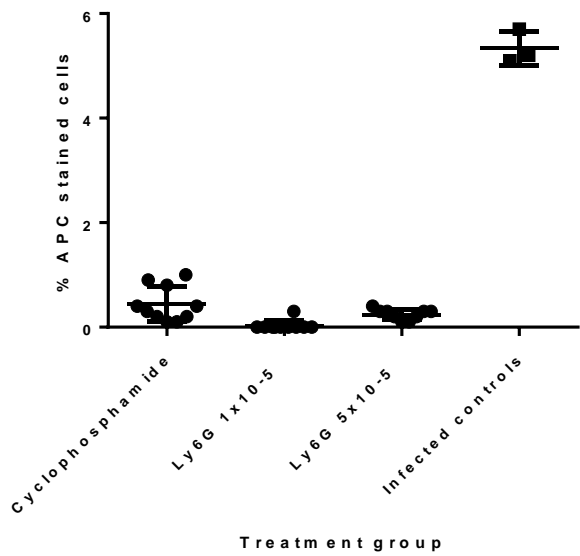

E.

Supplement: S6 Fig — (A) Representative liver sections from a control mouse, (B) Representative liver sections from a mouse treated with clodronate. Both sections were stained with antibody against the macrophage marker F4/80 (Biorad, MCA497) then stained with an anti-rat red fluorescent antibody. Few if any macrophages are visible in the clodronate treated mice. Scale bars: 50μm. (C) Representative flow cytometry from a non-treated control mouse. (D) Representative flow cytometry from a mouse treated with anti-Ly6G antibody. (E) Proportional neutrophil counts in Neutrophil depleted mice against a control group of non-treated mice (n- = 3). Very few neutrophils are detectable in treated groups compared to the infected controls, although some are present in the cyclophosphamide treated group. (PDF) [file ppat.1007112.s006.pdf]

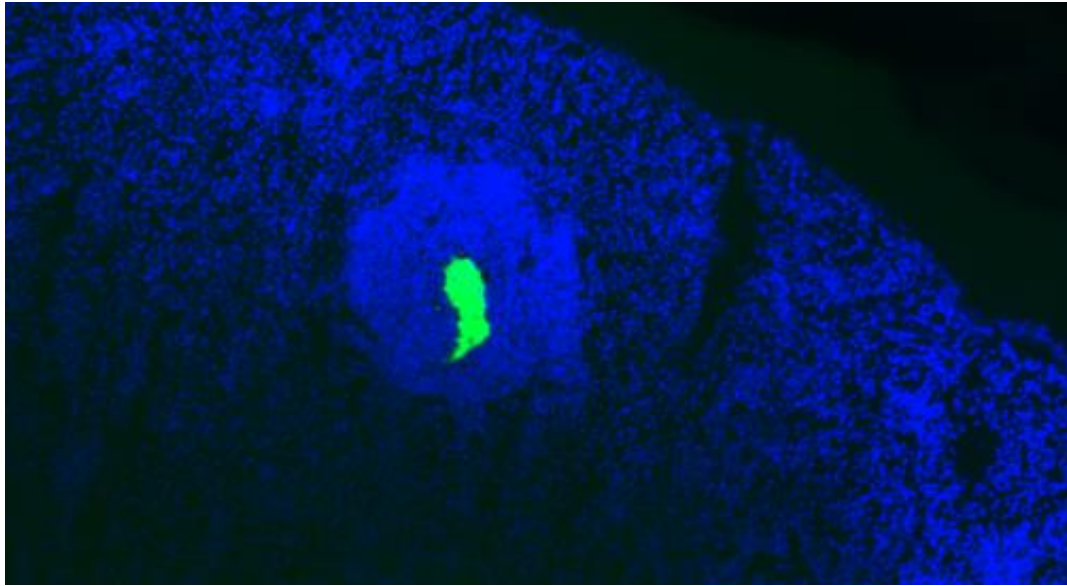

A.

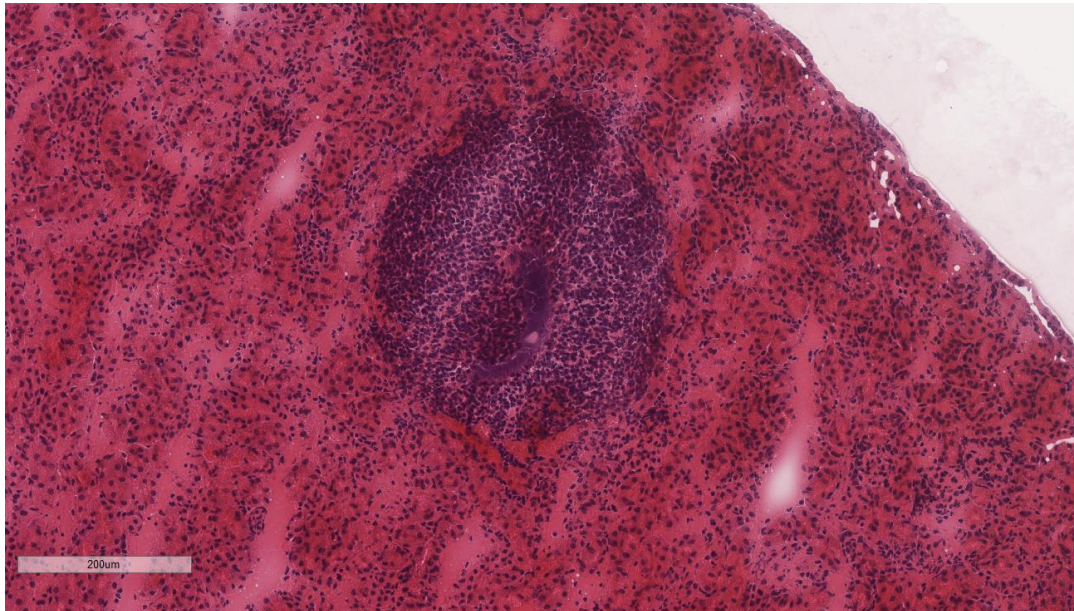

B.

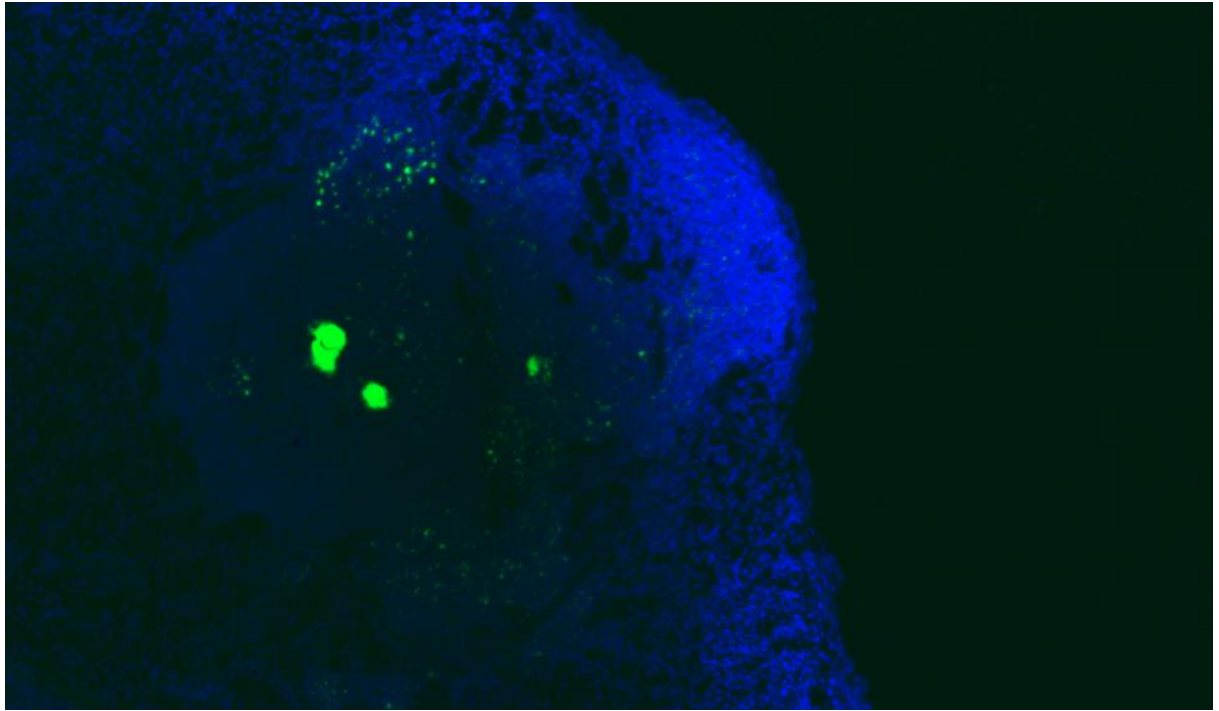

C.

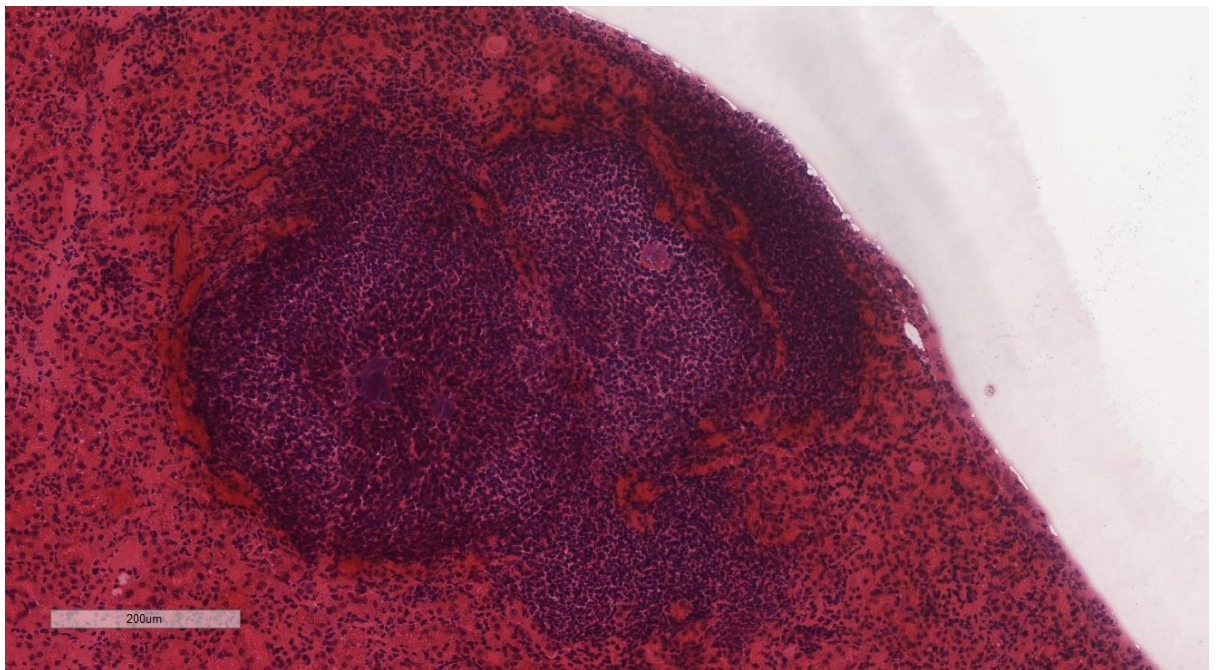

D.

Supplement: S7 Fig — Neutrophils are found surrounding S. aureus aggregates in abscesses as shown in serial sections. (A) One section showing a S. aureus GFP aggregate. (B) The serial section below stained with Haematoxylin and Eosin showing the neutrophils surrounding it. (C) Another section showing a mix of aggregates and diffuse groupings of S. aureus GFP. (D) The serial section below stained with Haematoxylin and Eosin showing the neutrophils surrounding these bacteria as well as fibrosis around the abscess. (PDF) [file ppat.1007112.s007.pdf]
