# Supplementary material for: Staphylococcus aureus infection dynamics
Source: PLoS Pathog. 2018 Jun 14;14(6):e1007112. doi: 10.1371/journal.ppat.1007112 (PMC6019756; doi:10.1371/journal.ppat.1007112)

A. SH1000 Sepsis model

Day of death	2hrs					18hrs					48hrs					72hrs				
Mouse number	1	2	3	4	5	6	7	8	9	10	11	12	13	14	15	16	17	18	19	20
Heart	–	–	–	–	–	–	–	2	–	–	–	–	–	–	–	–	–	–	–	–
Lungs	–	–	–	–	–	–	–	–	2	–	–	–	–	–	–	–	–	–	–	–
Spleen	5	–	–	5	–	–	4	4	4	3	–	–	–	–	–	–	–	2	–	–
Left Kidney	–	–	–	–	–	–	–	–	2	–	–	–	–	–	–	–	–	–	–	5
Right Kidney	–	–	–	–	–	–	–	–	–	–	–	–	–	–	–	3	–	2	–	6
Liver	6	6	6	6	6	6	5	5	5	5	3	4	4	4	4	3	3	4	3	3

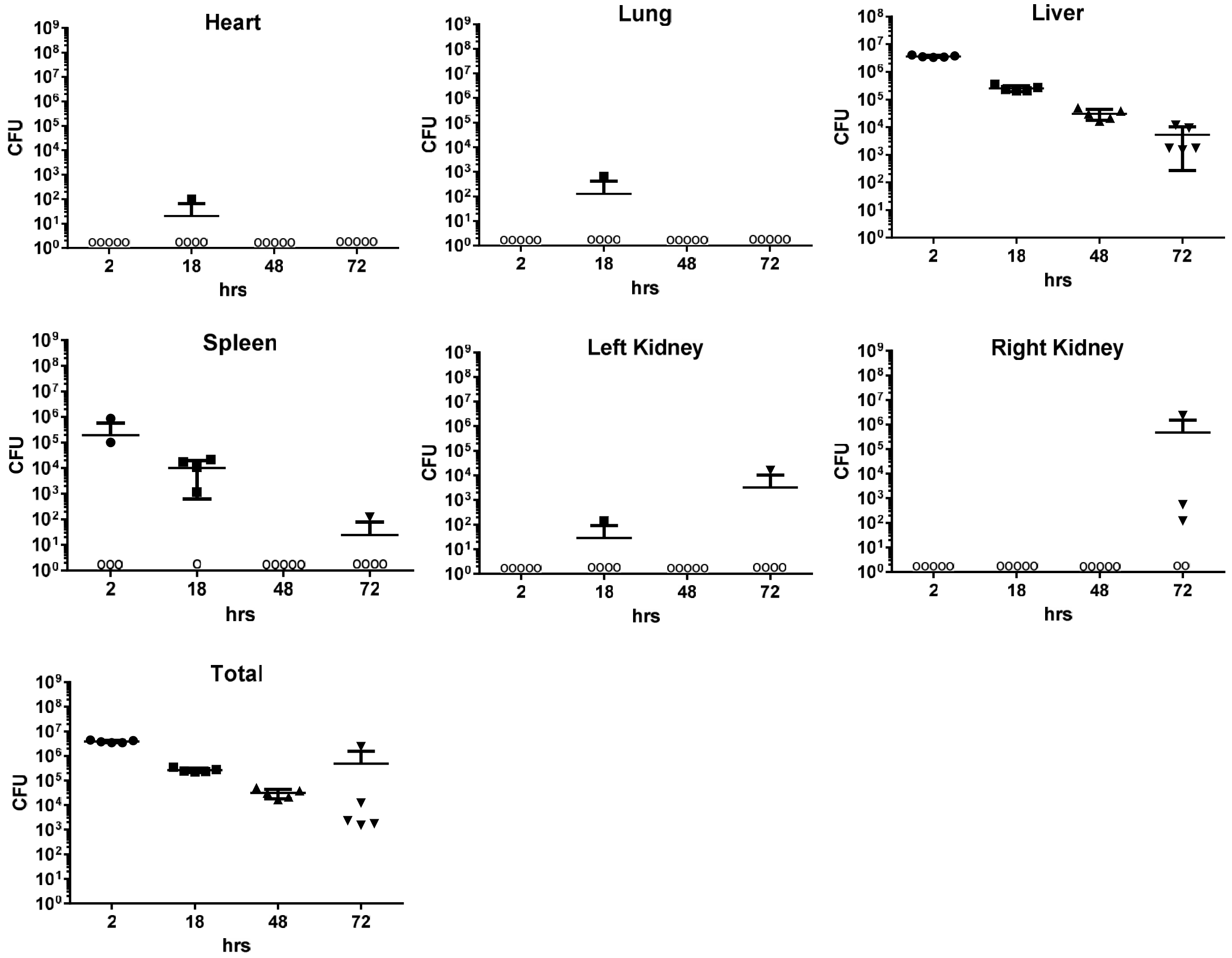

B. Newman Sepsis model

Day of death	2hrs					18hrs					48hrs					72hrs				
Mouse number	1	2	3	4	5	6	7	8	9	10	11	12	13	14	15	16	17	18	19	20
Heart	–	–	–	–	–	2	–	–	–	4	–	–	–	–	3	–	–	–	–	–
Lungs	–	–	–	–	–	–	–	–	–	–	–	–	–	–	–	–	–	–	–	–
Spleen	2	3	3	5	3	3	3	2	2	3	–	–	–	–	–	–	–	–	3	–
Left Kidney	–	–	–	–	–	2	–	–	–	–	–	–	–	–	–	–	–	–	3	–
Right Kidney	–	–	–	–	–	–	–	–	–	2	–	–	–	–	–	–	–	–	2	–
Liver	5	6	5	5	5	3	3	4	3	3	6	5	3	6	7	7	2	3	4	3

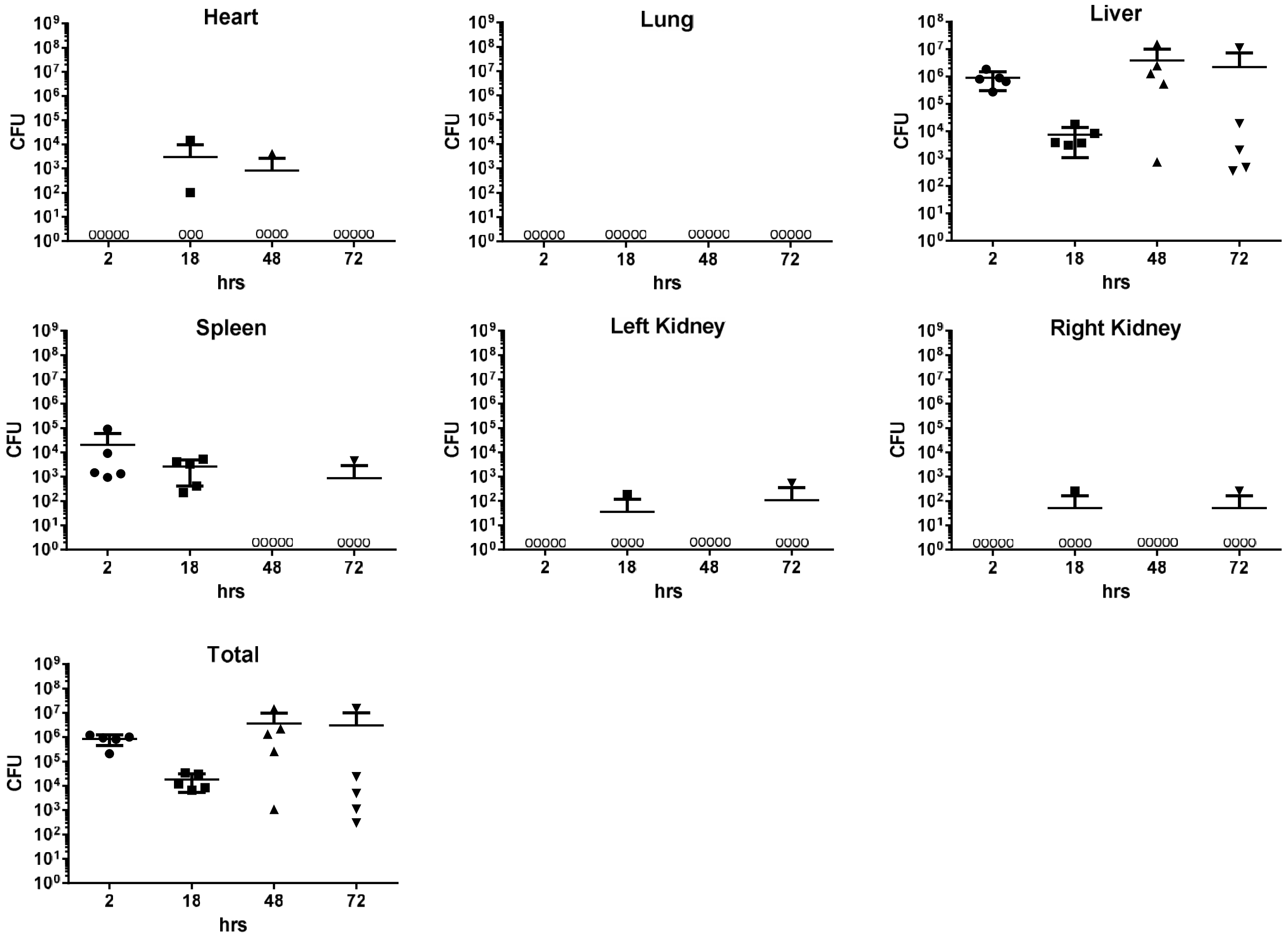

C. USA300 (JE2) Sepsis model

Day of death	2hrs					18hrs					48hrs					72hrs				
Mouse number	1	2	3	4	5	6	7	8	9	10	11	12	13	14	15	16	17	18	19	20
Heart	–	2	–	–	–	–	–	–	–	–	–	–	–	–	–	–	–	–	2	–
Lungs	–	–	–	–	–	–	–	–	–	–	–	–	–	–	–	–	–	–	–	–
Spleen	–	–	–	–	–	–	2	–	–	–	–	–	–	–	–	–	–	–	–	–
Left Kidney	–	–	–	2	–	–	4	–	2	5	3	6	4	2	–	5	2	5	–	3
Right Kidney	–	–	–	–	–	–	–	–	–	–	–	–	–	–	–	–	6	–	–	–
Liver	6	5	6	6	4	4	4	4	4	4	3	2	3	6	–	5	2	4	6	4

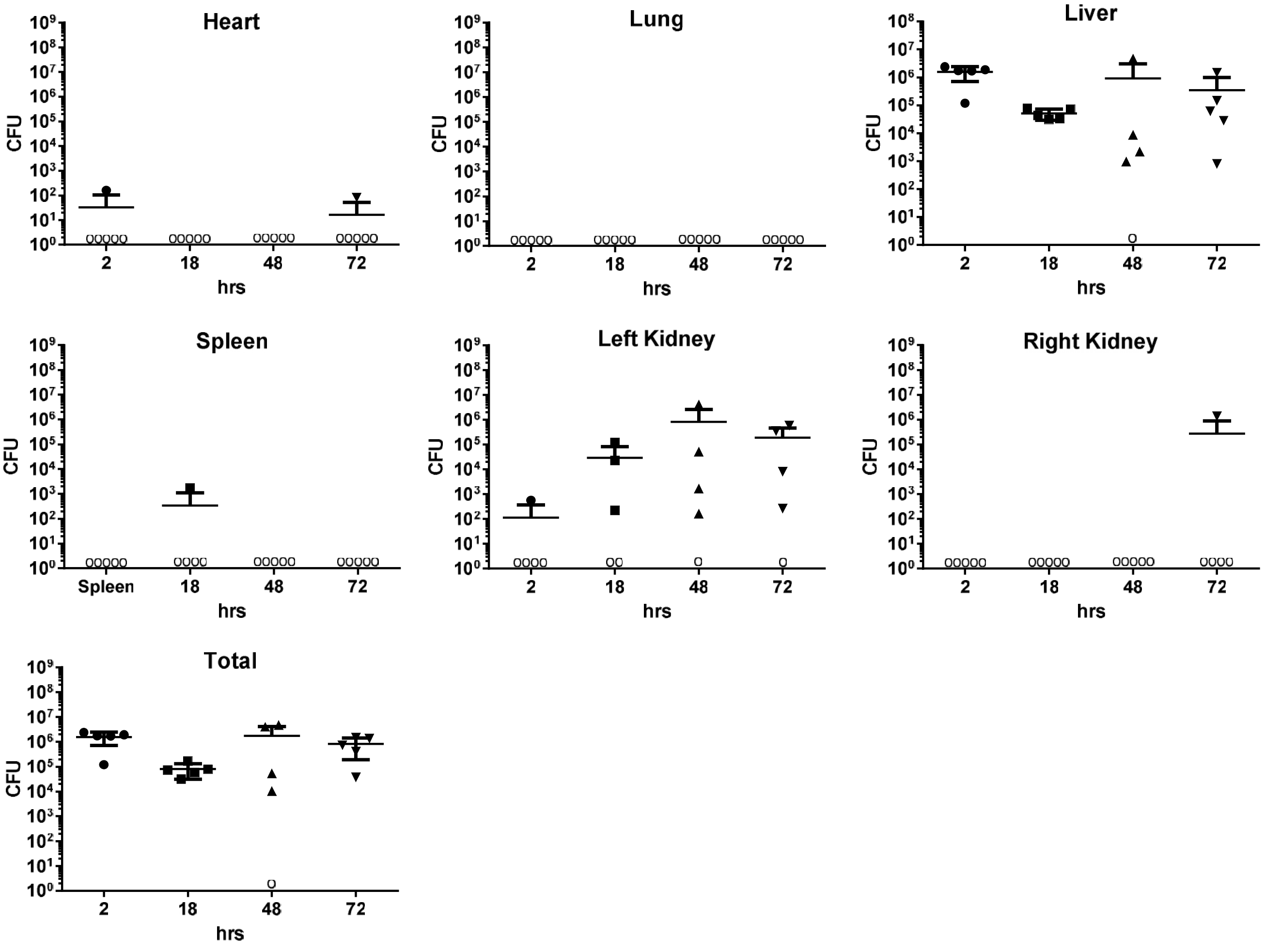

Supplement: S2 Fig — S. aureus distribution at different time points during the mouse sepsis model for SH1000 (A), Newman (B) and USA300 (C). Mice were infected with a 1:1:1 mixture of 3 resistance marker tagged variants for each strain. For each panel, above shows the proportions of each strain at each time point in the different organs in each mouse. The number in each represents the log amount of bacteria (e.g. 10−6 CFU = 6). Below shows the CFU load at each time point for the organs and total CFU. Organs with CFU counts below the limit of detection (<100CFU) are represented by open circles. Error bars: mean ± SD. 5 mice were sacrificed in each study at 2hrs, 18hrs, 48hrs and 72hrs post injection of S. aureus. (PDF) [file ppat.1007112.s002.pdf]
