# Supplementary material for: Staphylococcus aureus infection dynamics
Source: PLoS Pathog. 2018 Jun 14;14(6):e1007112. doi: 10.1371/journal.ppat.1007112 (PMC6019756; doi:10.1371/journal.ppat.1007112)

A. USA300 (JE2) Survival model

Day of death	Day 2				Day 3				Day 4			Day 5		Day 6		Day 11 (end of experiment)				
Mouse number	1	6	15	19	8	10	12	18	14	17	20	3	16	7	13	2	4	5	9	11
Heart	4	–	–	7	4	7	7	7	–	–	–	–	3	–	4	2	4	–	–	3
Lungs	5	–	–	–	–	5	2	2	–	–	–	3	2	2	2	–	5	–	–	2
Spleen	3	4	3	5	5	–	–	5	–	–	2	–	–	–	–	–	4	–	–	–
Left Kidney	2	–	5	6	5	6	8	5	6	5	8	5	7	5	5	7	6	8	6	7
Right Kidney	7	6	–	3	4	8	7	–	2	2	2	4	7	7	6	4	7	5	6	5
Liver	7	7	7	7	8	7	8	8	4	7	3	3	2	7	2	–	7	3	–	3

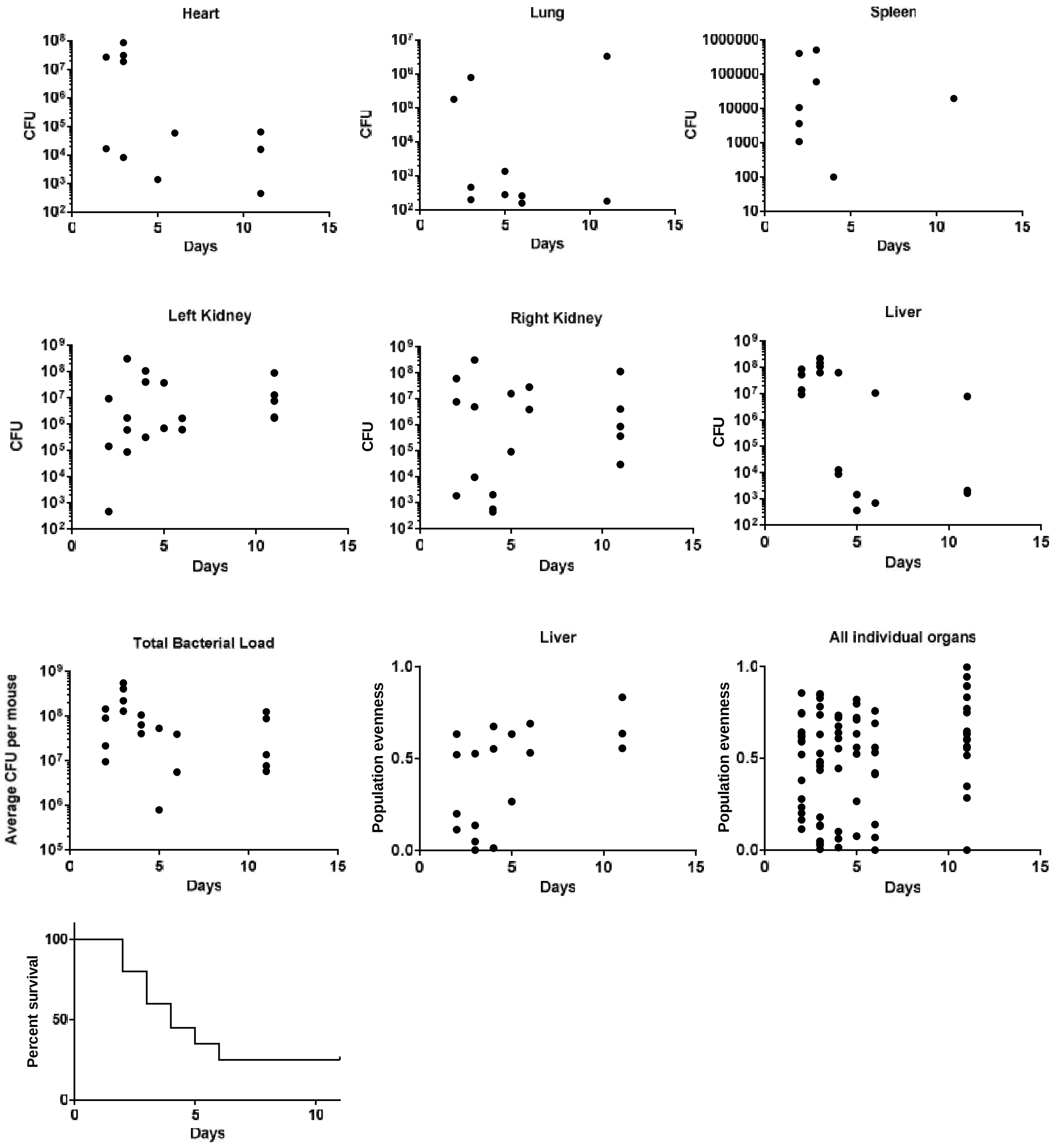

B. Newman Survival model

Day of death	Day 2	Day 3								Day 4								Day 5		
Mouse number	20	3	4	5	10	12	13	17	18	6	7	8	9	11	14	16	19	1	2	15
Heart	3	8	5	7	7	7	7	6	6	6	5	5	6	6	–	4	7	5	3	–
Lungs	–	–	–	–	–	–	–	–	3	3	–	–	–	–	–	–	5	2	2	–
Spleen	3	4	3	4	4	4	2	4	3	3	4	–	5	2	–	2	4	5	4	2
Left Kidney	6	5	8	7	6	5	9	6	7	4	7	5	7	5	5	8	7	7	6	6
Right Kidney	6	5	8	5	7	6	8	3	8	5	7	5	7	5	6	8	6	7	6	6
Liver	7	8	7	7	7	7	7	7	7	7	7	8	7	7	7	7	7	7	8	8

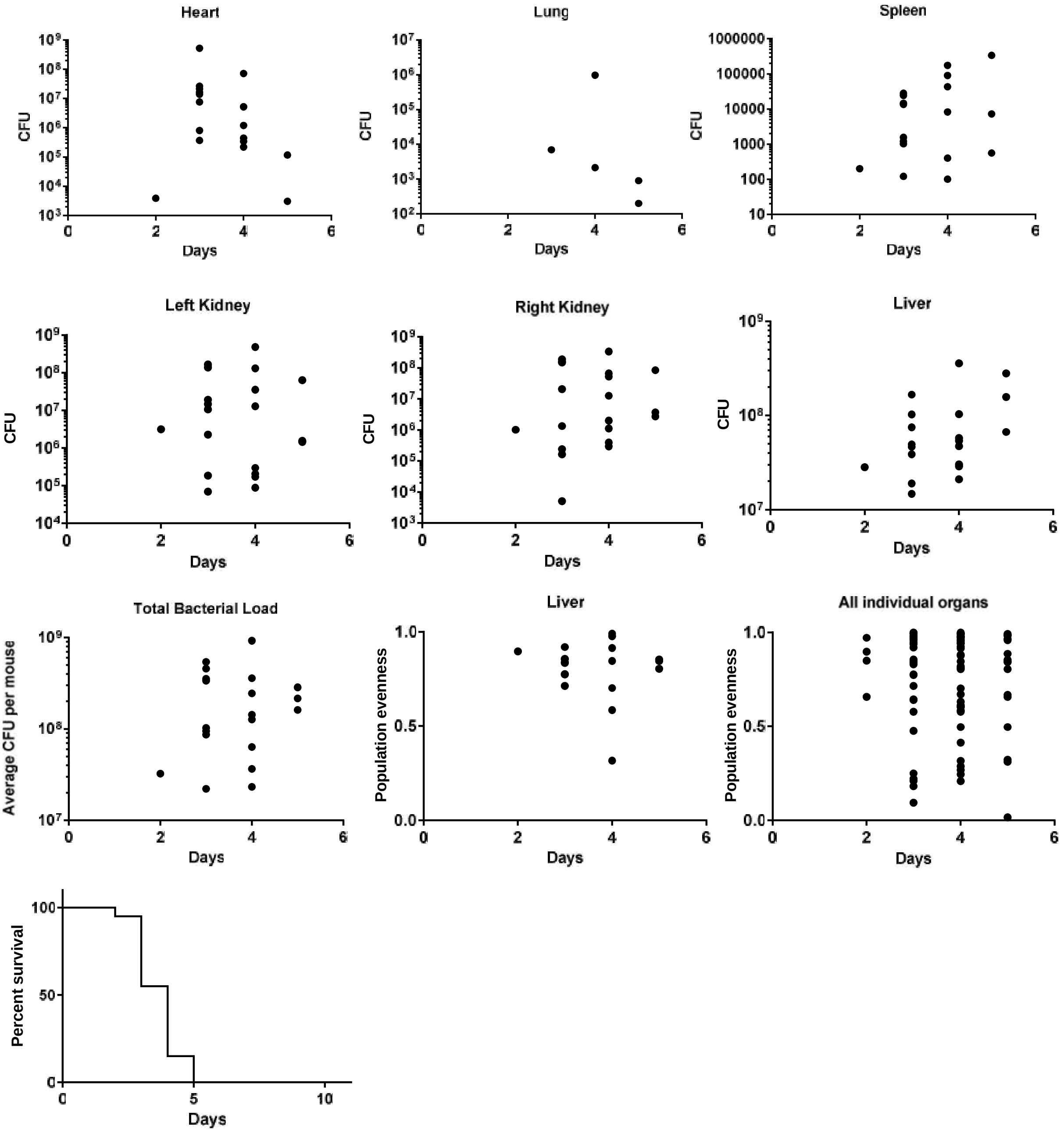

C. SH1000 Survival model

Day of death	Day 1	Day2				Day3			Day4				Day5		Day6		Day8	Day 11(end of experiment)		
Mouse number	14	8	10	11	20	6	17	18	2	3	13	15	7	12	4	16	5	1	9	19
Heart	–	6	–	–	–	–	–	–	3	6	7	6	6	–	5	–	5	2	–	–
Lungs	2	3	5	–	2	–	2	–	2	3	6	3	5	2	5	3	5	3	–	3
Spleen	–	–	–	–	2	–	2	–	–	–	4	–	2	–	2	3	–	2	–	–
Left Kidney	–	–	–	4	–	–	–	2	2	3	2	–	3	3	–	–	–	–	–	–
Right Kidney	6	7	2	6	3	–	2	–	7	5	–	3	6	7	5	–	7	4	5	5
Liver	7	7	3	7	7	6	7	5	5	8	7	6	7	6	7	3	4	5	4	5

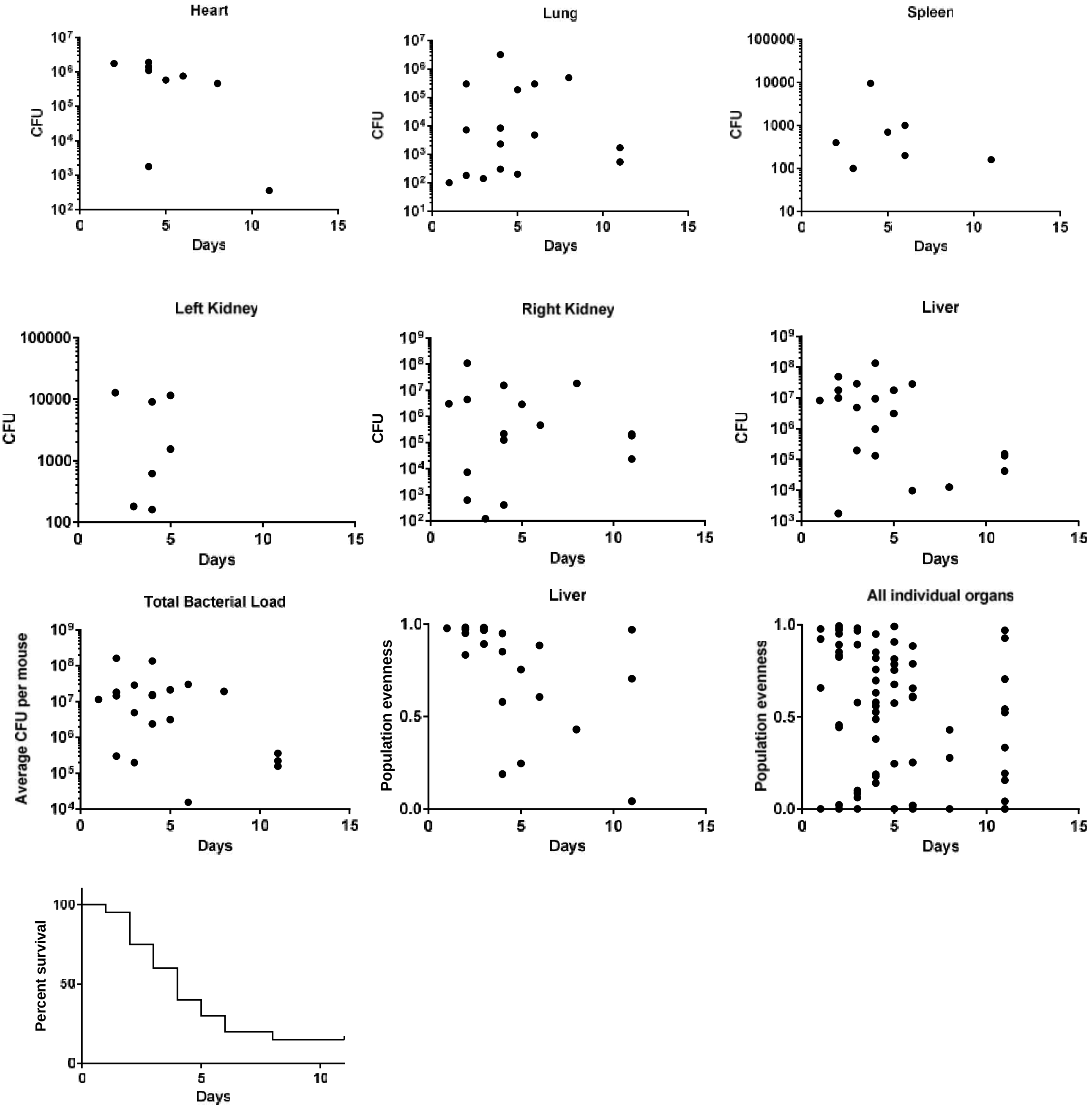

Supplement: S3 Fig — S. aureus distribution at different time points during the mouse survival model for USA300 (A), Newman (B) and SH1000 (C). Mice were infected with a 1:1:1 mixture of 3 resistance marker tagged variants and 5 mice sacrificed as they reached the severity limits. For each panel, above is shown the proportions of each strain at each time point in the different organs in each mouse. The number in each represents the log amount of bacteria (e.g. 10−6 CFU = 6). Below is shown the CFU load at each time point for the organs and total CFU as well as the survival curve. The population evenness of the liver and all the individual organs is also shown. For the USA300 study, on each day the following numbers of mice were sacrificed due to reaching severity limits: Day 2:4, Day 3:4, Day 4:3, Day 5:2, Day 6:2, Day 11: 5 (end of procedure). For the Newman study, on each day the following numbers of mice were sacrificed due to reaching severity limits: Day 2:1, Day 3:8, Day 4:8, Day 5:3. For the SH1000 study, on each day the following numbers of mice were sacrificed due to reaching severity limits: Day 1:1, Day 2:4, Day 3:3, Day 4:4, Day 5:2, Day 6: 2, Day 11: 4 (end of procedure). (PDF) [file ppat.1007112.s003.pdf]
