# Supplementary material for: Staphylococcus aureus infection dynamics
Source: PLoS Pathog. 2018 Jun 14;14(6):e1007112. doi: 10.1371/journal.ppat.1007112 (PMC6019756; doi:10.1371/journal.ppat.1007112)

A. NewHG Lower dose survival model

Day of death	Day 2				Day 3			Day 4		Day 5			8	9	10	Day 11				
Mouse number	1	3	6	12	14	19	20	5	16	4	15	18	8	13	17	2	7	9	10	11
Heart	5	3	–	–	–	–	6	–	–	4	–	–	–	–	–	–	–	–	–	–
Lungs	5	2	–	3	–	2	–	7	2	6	–	–	–	–	–	–	–	–	–	–
Spleen	6	3	–	–	–	–	6	–	2	5	–	–	–	–	–	–	–	–	–	–
Left Kidney	7	5	5	5	7	7	6	7	6	7	5	7	7	4	7	5	5	–	–	5
Right Kidney	7	6	6	5	4	–	–	7	9	7	7	7	6	7	6	–	6	–	–	5
Liver	8	8	7	8	5	3	8	7	3	6	6	6	–	–	–	2	3	–	–	–

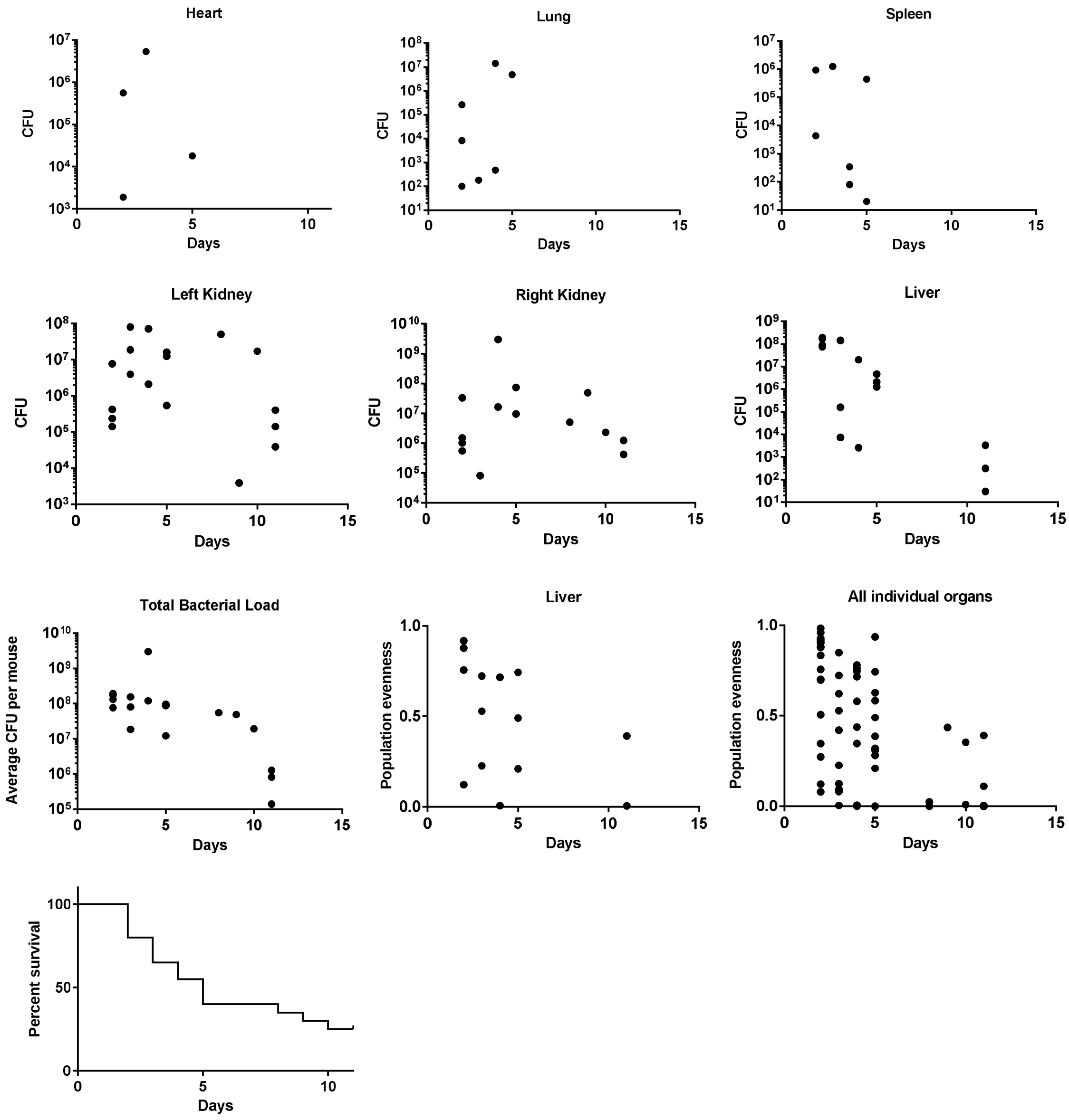

B. SH1000 Lower dose survival model

Day of death	4	6	8	11 days (end of experiment)																
Mouse number	9	13	18	1	2	3	4	5	6	7	8	10	11	12	14	15	16	17	19	20
Heart	–	–	–	–	–	–	–	–	–	–	–	–	–	–	–	–	–	–	–	–
Lungs	–	–	–	–	–	–	–	–	–	–	–	–	–	–	–	–	–	–	–	–
Spleen	2	–	–	–	–	–	–	–	–	–	–	–	–	–	–	–	–	–	–	–
Left Kidney	5	7	5	–	–	–	–	–	5	7	4	4	–	–	–	–	–	–	–	–
Right Kidney	4	5	–	7	–	–	–	–	–	4	–	4	–	–	–	–	4	–	–	–
Liver	4	2	2	3	3	4	–	2	2	2	–	–	–	4	2	–	–	–	–	–

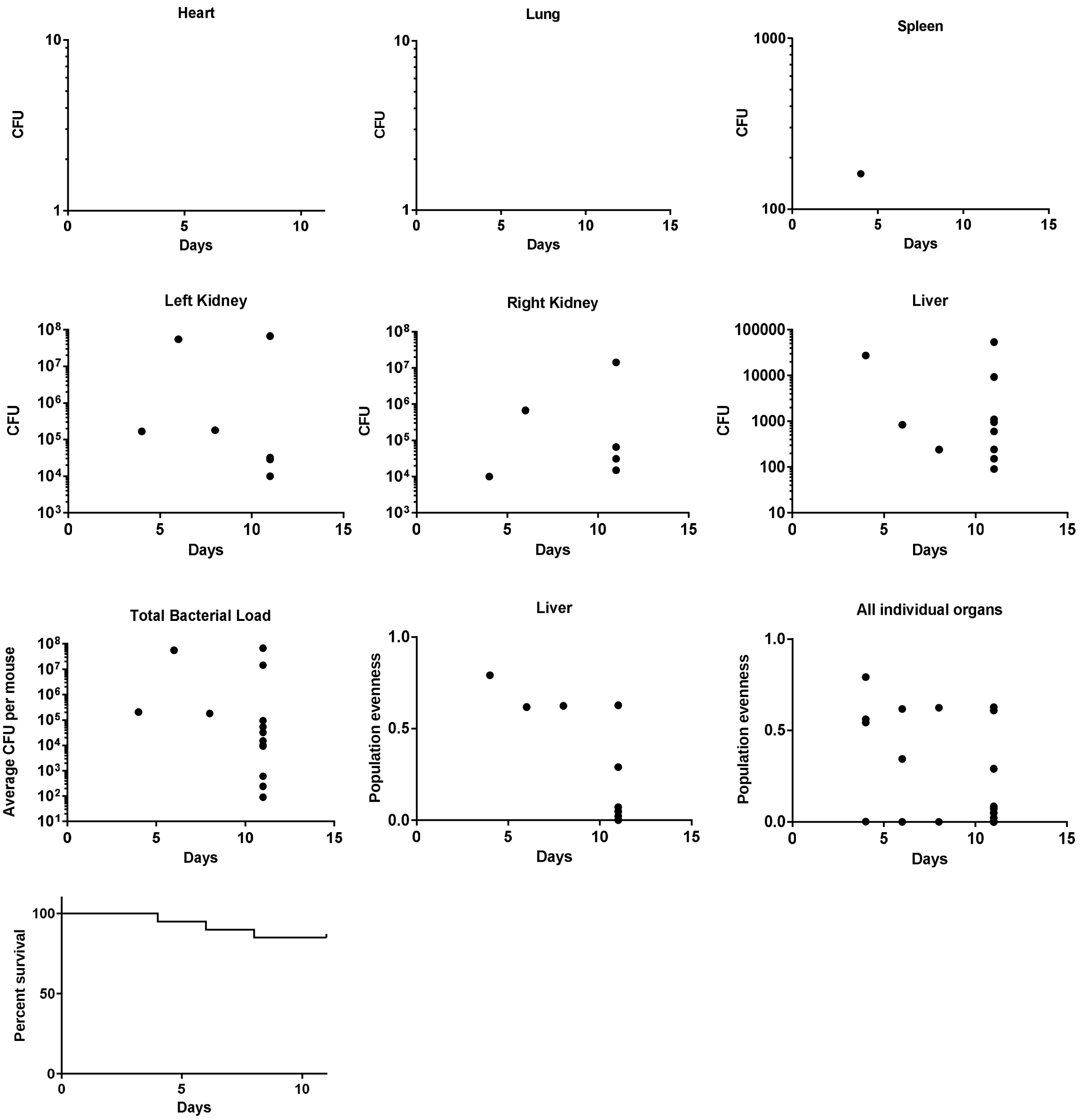

C. USA300 (JE2) Lower dose survival model

Day of death	Day 2		Day 4		Day 11(end of experiment)															
Mouse number	1	20	14	17	2	3	4	5	6	7	8	9	10	11	12	13	15	16	18	19
Heart	4	7	–	–	–	–	–	–	–	–	–	–	–	–	–	–	–	–	–	–
Lungs	–	–	–	–	–	–	–	–	–	–	–	–	–	–	–	–	–	–	–	–
Spleen	–	–	–	–	–	–	–	–	–	–	–	–	–	–	–	–	–	–	–	–
Left Kidney	–	–	2	–	–	–	2	–	3	–	–	2	2	–	2	–	–	–	–	–
Right Kidney	–	–	–	–	–	–	2	–	2	–	2	4	–	–	–	–	–	–	–	–
Liver	4	8	4	4	–	–	2	4	3	3	–	2	–	3	5	–	–	–	–	–

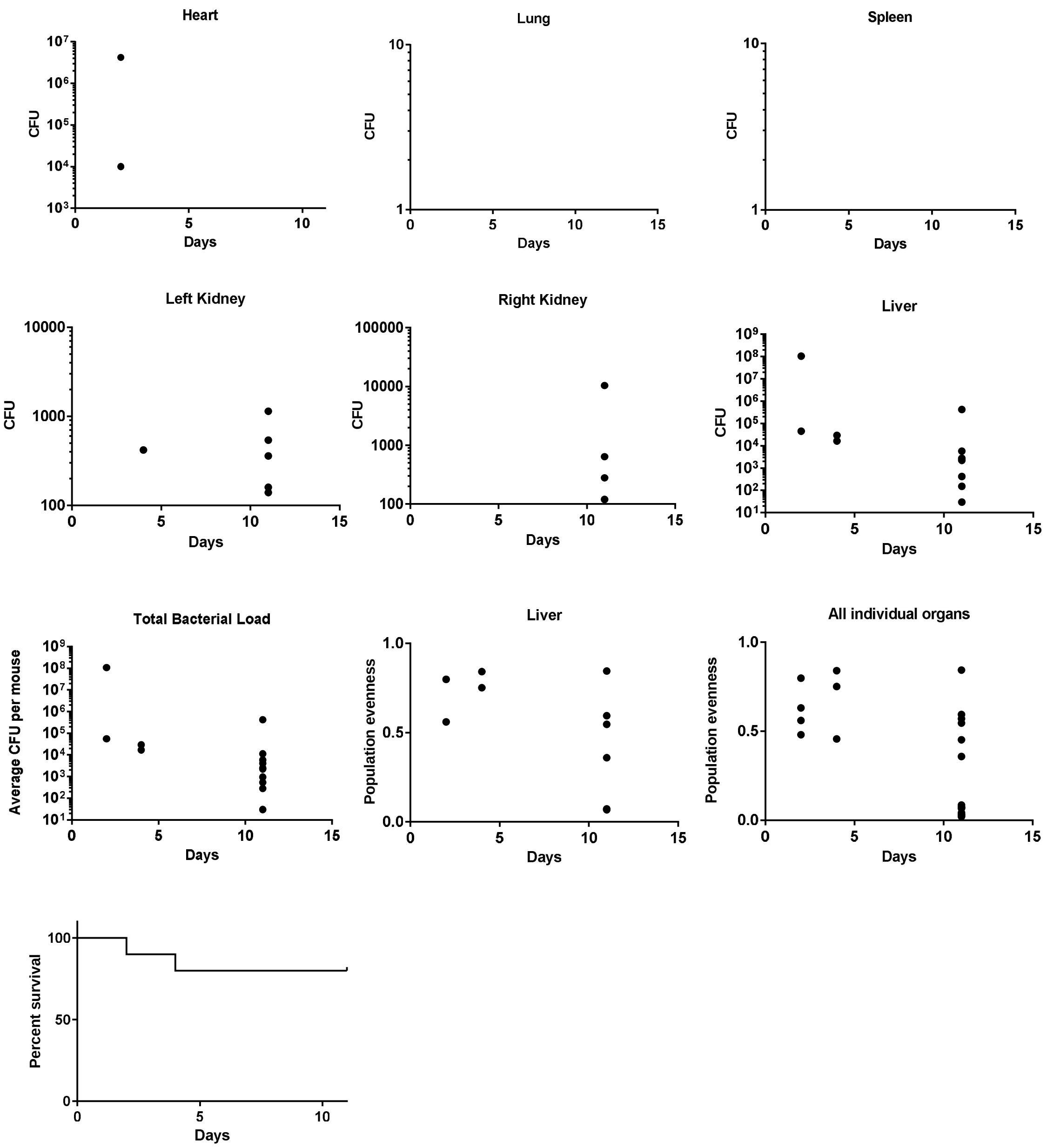

D. Newman Lower dose survival model

Day of death	Day 3			Day 4		Day 5					Day 6		Day 7	Day 8			Day 10	Day 11 (end of experiment)		
Mouse number	2	14	18	1	20	4	7	11	12	15	8	9	17	5	10	19	6	3	13	16
Heart	–	–	–	7	–	–	–	–	–	–	–	–	–	–	–	–	–	–	–	–
Lungs	–	–	–	–	–	–	–	–	–	–	–	–	–	–	–	–	–	–	–	–
Spleen	–	5	–	5	–	–	–	–	–	–	–	–	–	–	–	–	–	–	–	–
Left Kidney	–	–	6	–	–	–	6	6	–	6	6	–	–	–	–	–	5	–	5	–
Right Kidney	2	4	–	2	–	3	3	6	3	4	8	7	3	4	4	–	6	–	3	–
Liver	6	7	7	7	7	7	7	7	8	7	7	8	7	5	8	8	7	7	8	7

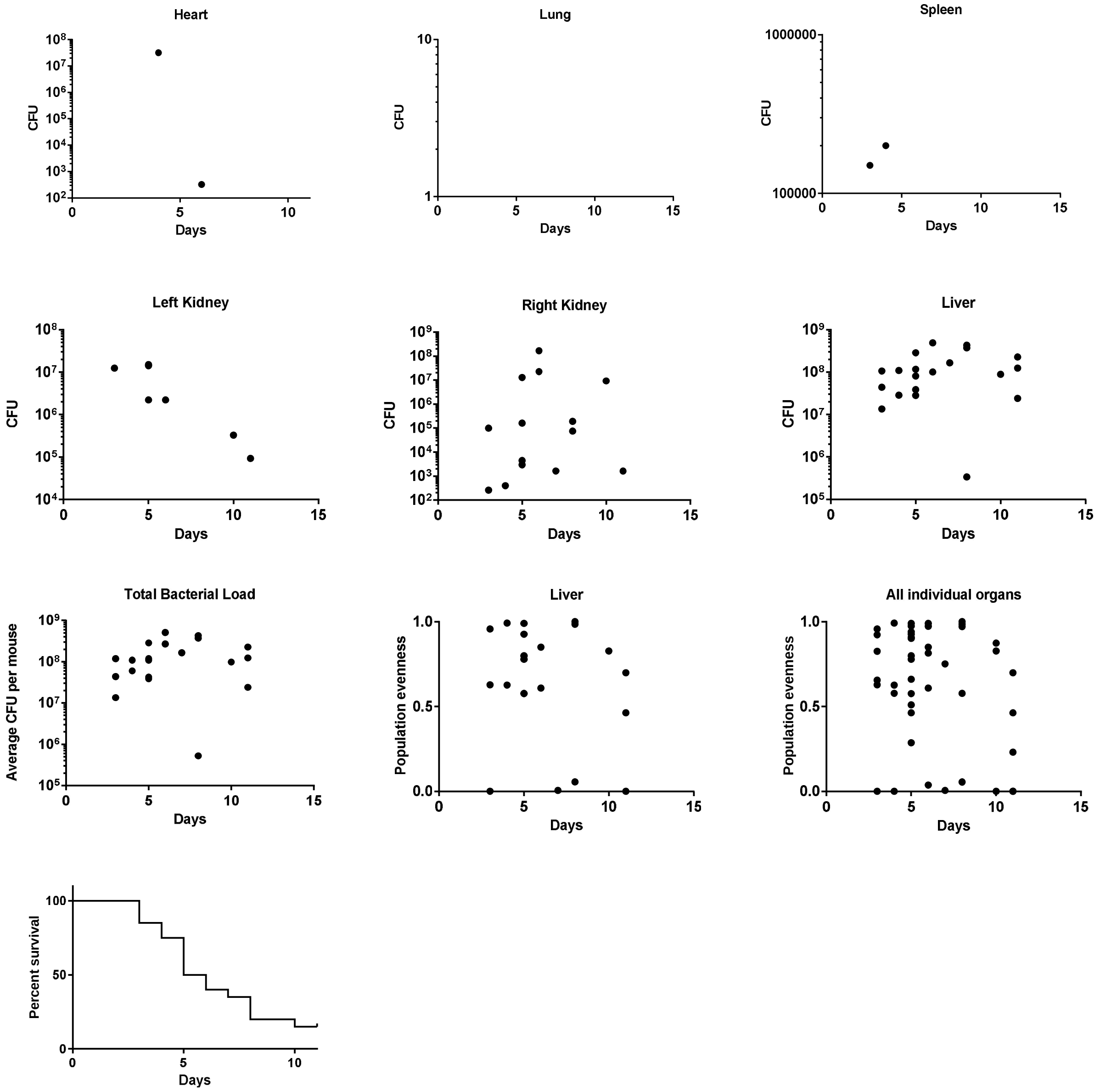

Supplement: S4 Fig — S. aureus distribution at different time points during the mouse survival model (lower dose) for NewHG (A), SH1000 (B), USA300 (C) and Newman (D). Mice were infected with a 1:1:1 mixture of 3 resistance marker tagged variants and 5 mice sacrificed as they reached the severity limits. Here the mice were given a substantially lower dose than in the other survival studies. For each panel, above is shown the proportions of each strain at each time point in the different organs in each mouse. The number in each represents the log amount of bacteria (e.g. 10−6 CFU = 6). Below is shown the CFU load at each time point for the organs and total CFU as well as the survival curve. The population evenness of the liver and all the individual organs is also shown. For the NewHG study, the following numbers of mice were sacrificed due to reaching severity limits: Day 2:4, Day 3:3, Day 4:2, Day 5:3, Day 8:1, Day 9:1, Day 10:1, Day 11:5 (end of procedure). For the Newman study, the following numbers of mice were sacrificed due to reaching severity limits: Day 3:3, Day 4:2, Day 5:5, Day 6:2, Day 7:1, Day 8:3, Day 10:1, Day 11: 3 (end of procedure). For the USA300 study, on each day the following numbers of mice were sacrificed due to reaching severity limits: Day 2:2, Day 4:2, Day 11: 16 (end of procedure). For the SH1000 study, on each day the following numbers of mice were sacrificed due to reaching severity limits: Day 4:1, Day 6:1, Day 8:1, Day 11: 17 (end of procedure). (PDF) [file ppat.1007112.s004.pdf]
