# Supplementary material for: Staphylococcus aureus infection dynamics
Source: PLoS Pathog. 2018 Jun 14;14(6):e1007112. doi: 10.1371/journal.ppat.1007112 (PMC6019756; doi:10.1371/journal.ppat.1007112)
Supplement: S5 Fig — (A) The proportions of each strain in the various organs in control mice (blank liposomes) injected with 1x10^5^ CFU (counts and diversity shown in Fig 4A and 4B). (B) The proportions of each strain in the various organs in mice injected with 1x10^5^ CFU and clodronate. In a repeat of the previous study shown in Fig 4, mixed populations occur in the liver and spread to other organs occurs. (C) The proportions of each strain in the various organs in mice injected with 1x10^5^ CFU and anti Ly-6G. Again the liver populations are largely clonal and there is no spread to other organs. However the numbers were equivalent to the blank controls so consequently the dose was increased the dose for the depletion study (Fig 4). (D) The proportions of each strain in the various organs in mice injected with 1x10^5^ CFU and cyclophosphamide. There are higher loads in the livers and clonality occurs. All mice were sacrificed 3 days post infection apart from the cyclophosphamide study where the mice were sacrificed due to hitting severity limits as follows: Day 1:2, Day 2:5, Day 3: 3 (end of procedure). (E) The CFU in individual livers. (F) The population evenness of the bacteria in individual livers. Error bars: mean ± SD. (PDF) [file ppat.1007112.s005.pdf]

	1x10-5 + blank Liposomes									
Mouse number	1	2	3	4	5	6	7	8	9	10
Heart	–	–	–	–	–	–	–	–	–	–
Lungs	–	–	–	–	–	–	–	–	–	–
Spleen	–	–	–	–	–	–	–	–	–	–
Left Kidney	–	–	–	–	–	–	–	–	–	–
Right Kidney	–	–	–	–	–	–	–	–	–	–
Liver	2	2	3	3	3	2	–	3	2	2

A.

	1x10-5 + Clodronate (repeat)									
Mouse number	1	2	3	4	5	6	7	8	9	10
Heart	–	3	–	4	–	–	–	–	–	2
Lungs	–	–	–	–	–	–	–	–	–	–
Spleen	–	–	–	–	–	–	2	–	–	–
Left Kidney	–	2	–	–	–	–	–	–	6	3
Right Kidney	2	–	–	–	–	2	–	4	2	3
Liver	7	7	8	8	7	8	8	8	7	7

B.

	1x10-5 + Anti Ly6G								
Mouse number	1	2	3	4	5	6	7	8	9
Heart	–	–	–	–	–	–	–	–	–
Lungs	–	–	–	–	–	–	–	–	–
Spleen	–	–	–	–	–	–	–	–	–
Left Kidney	–	–	–	–	–	–	–	–	–
Right Kidney	–	–	–	–	–	–	–	–	–
Liver	4	3	–	3	2	3	–	2	2

C.

	1x10-5 + Cyclophosphamide									
Day of death	3	2	2	1	3	1	2	2	3	2
Mouse number	1	2	3	4	5	6	7	8	9	10
Heart	–	3	–	–	–	–	–	–	3	–
Lungs	–	–	–	–	–	–	–	–	–	–
Spleen	–	–	–	3	2	–	–	–	–	–
Left Kidney	–	–	–	–	–	–	–	–	2	–
Right Kidney	–	–	–	–	–	–	–	–	–	–
Liver	–	6	7	4	6	3	6	5	6	7

D.

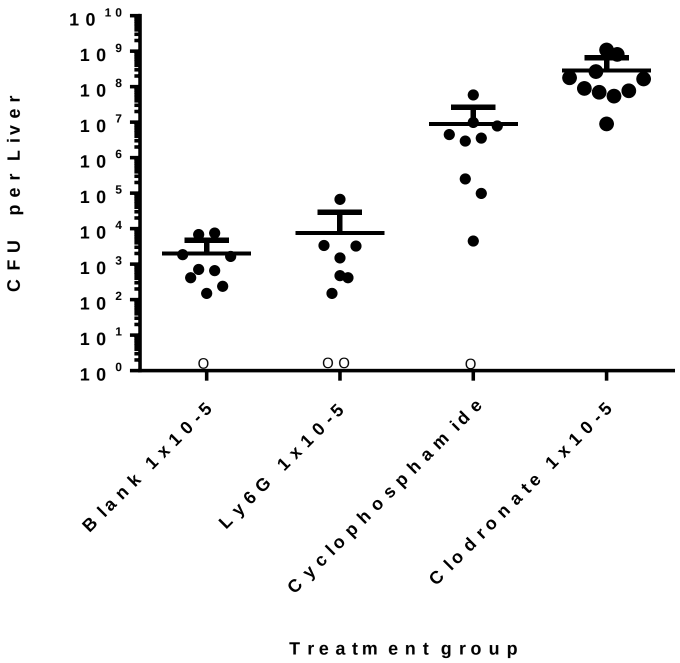
CFU per Liver
10^{10}
10^{9}
10^{8}
10^{7}
10^{6}
10^{5}
10^{4}
10^{3}
10^{2}
10^{1}
10^{0}
Blank 1x10-5
Ly6G 1x10-5
Cyclophosphamide
Clodronate 1x10-5
Treatment group

E.

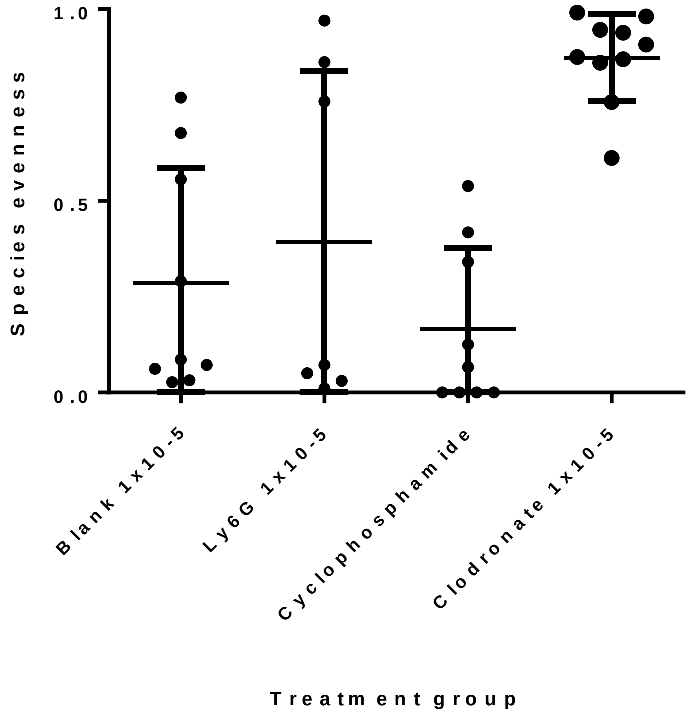
Species evenness
1.0
0.5
0.0
Blank 1x10-5
Ly6G 1x10-5
Cyclophosphamide
Clodronate 1x10-5
Treatment group

F.
