# Supplementary material for: Staphylococcus aureus infection dynamics
Source: PLoS Pathog. 2018 Jun 14;14(6):e1007112. doi: 10.1371/journal.ppat.1007112 (PMC6019756; doi:10.1371/journal.ppat.1007112)
Supplement: S1 Table — (PDF) [file ppat.1007112.s009.pdf]

Table S1.Bacterial strains and plasmids used in this study.

Strain/Plasmid	Description	Reference
SH1000	Functional *rsbU+* derivative of *S. aureus* 8325-4	(34)
RN4220	Restriction-deficient transformation recipient *S. aureus*	(52)
Newman	Common laboratory strain used in animal studies	(33)
NewHG	*S. aureus* Newman with *saeS*^{L} allele from strain RN1	(53)
USA300 (JE2)	Plasmid cured USA300 strain, parent strain of the Nebraska transposon library	(35)
GMSA015	SH1000 *lysA*::pGM068 (EryR) *lysA+*	(11)
GMSA016	SH1000 *lysA*::pGM072 (KanR) *lysA+*	(11)
GMSA017	SH1000 *lysA*::pGM070 (TetR) *lysA+*	(11)
GMSA021	NewHG *lysA*::pGM068 (EryR) *lysA+*	(11)
GMSA022	NewHG *lysA*::pGM072 (KanR) *lysA+*	(11)
GMSA023	NewHG *lysA*::pGM070 (TetR) *lysA+*	(11)
EPPS1	JE2 *lysA*::pGM068 (EryR) *lysA+*	This study
EPPS2	JE2 *lysA*::pGM072 (KanR) *lysA+*	This study
EPPS3	JE2 *lysA*::pGM070 (TetR) *lysA+*	This study
EPPS4	Newman *lysA*::pGM068 (EryR) *lysA+*	This study
EPPS5	Newman *lysA*::pGM072 (KanR) *lysA+*	This study
EPPS6	Newman *lysA*::pGM070 (TetR) *lysA+*	This study
EP01	RN4220 GFP KanR	This study
EP02	NewHG GFP KanR	This study
PS01	RN4220 mCherry TetR	This study
PS02	NewHG mCherry TetR EryR	This study
pEP01	pKasbar KanR GFP	This study
PEP02	pKasbar TetR GFP	This study
